# Item Response Thresholds Models: A General Class of Models for Varying Types of Items

**DOI:** 10.1007/s11336-022-09865-7

**Published:** 2022-04-27

**Authors:** Gerhard Tutz

**Affiliations:** Ludwig-Maximilians-Universität München, Akademiestraße 1, 80799 Munich, Germany

**Keywords:** thresholds model, latent trait models, item response theory, graded response model, Rasch model

## Abstract

A comprehensive class of models is proposed that can be used for continuous, binary, ordered categorical and count type responses. The difficulty of items is described by difficulty functions, which replace the item difficulty parameters that are typically used in item response models. They crucially determine the response distribution and make the models very flexible with regard to the range of distributions that are covered. The model class contains several widely used models as the binary Rasch model and the graded response model as special cases, allows for simplifications, and offers a distribution free alternative to count type items. A major strength of the models is that they can be used for mixed item formats, when different types of items are combined to measure abilities or attitudes. It is an immediate consequence of the comprehensive modeling approach that allows that difficulty functions automatically adapt to the response distribution. Basic properties of the model class are shown. Several real data sets are used to illustrate the flexibility of the models

**Supplementary Information:**

The online version contains supplementary material available at 10.1007/s11336-022-09865-7.

Modern item response theory provides a variety of models for the measurement of abilities, skills or attitudes, see, for example, Lord and Novick (1968), Van der Linden (2016a), Mair (2018). The history of its evolution has been traced back carefully by Van der Linden (2016b) and Thissen and Steinberg (2020).

Essential components of item response theory are that items can be located on the same scale as the latent trait and that the latent trait accounts for observed interrelationships among the item responses (Thissen and Steinberg, 2020). In addition, it is essential that the responses are random and have to be described by a probabilistic model to explain their distributions (Van der Linden, 2016b). These features distinguish item response theory from classical test theory (Lord and Novick, 1968), which uses an a priori score on the entire test by assuming an additive decomposition of an observed test score into a true score and a random error.

Item response models are typically tailored to the type of item. For binary items Rasch models and normal-ogive models are in common use (Rasch, 1961; Birnbaum, 1986), for ordered models the graded response model (Samejima, 1995, 2016), the partial credit model (Masters, 1982, Glas & Verhelst, 1989) and the sequential model (Tutz, 1989) have been used. For count data items, among others, Rasch’s Poisson count model and extensions as the Conway–Maxwell–Poisson model (Rasch, 1960; Forthmann et al., 2020) have been proposed. Continuous response models have been considered by Samejima (1973), Müller (1987), and Mellenbergh (2016). For taxonomies of item response models see Thissen and Steinberg (1986) and Tutz (2020).

The threshold model proposed here advances a unifying approach. Rather than developing different models for different types of responses a common response model for all sorts of responses is considered. In the model, each item has its own item difficulty function that determines the distribution of the response. Since item difficulty functions are item-specific, the form of the distribution can vary across items. The model class is rather general, it comprises various commonly used models as the binary Rasch model, the normal-ogive model and the graded response model, for the latter it offers a sparser parameterization. It also provides a genuine latent trait model for continuous responses, which can be seen as a latent trait version of classical test theory. In addition to providing a common framework for existing and novel models, it offers a way to combine different types of items in one test, what has been described as mixed item-formats. Instead of using linkage methods (Kim and Lee, 2006; Kolen and Brennan, 2014) to combine different items, the model itself accounts for the different sorts of items.

Major advantages of the approach are:

- The model provides a common framework for several models in common use.
- A genuine latent trait model for continuous responses as an alternative to classical test theory is contained as a special case.
- The model is very flexible and allows for quite different response distributions.
- Items can have different formats, they can be continuous, binary or polytomous, and the common model automatically accounts for the distributional differences. The model links performance on items that can differ in distributional form to person abilities.

The threshold model and basic concepts are introduced in Sect. 1. It is in particular demonstrated how difficulty functions can be used to model the distribution of responses. In Sect. 2, the case of discrete responses is considered and it is shown that common binary models and the graded response model are special cases of threshold models. In Sect. 3, further properties and alternative modeling approaches are considered. Section 4 is devoted to mixed item formats. In Section 5, a more flexible way of specifying difficulty functions is given, which allows to let the data determine which function fits best. The computation of estimates is considered in Sect. 6, although illustrative applications are given already in the previous sections. In the “Appendix”, results that are mentioned in the text are given in a more formal way together with proofs.

## Thresholds Models: Basic Concepts

Let $Y_{pi}$ denote the response of person *p* on item *i* ($p \in \{1,\dots ,P\}, i \in \{1,\dots ,I\}$) having support *S*. The general thresholds model we propose is given by

1 $$\begin{aligned} P(Y_{pi} > y|\theta _p,\alpha _i,\delta _{i}(.))=F(\alpha _i(\theta _p-\delta _{i}(y))), \end{aligned}$$

where *F*(.) is a strictly monotonically increasing distribution function, $\theta _p$ is a person parameter, $\alpha _i$ a discrimination parameter, and $\delta _{i}(.)$ is a non-decreasing item-specific function, called *item difficulty* function, which is defined on the support *S*. The function *F*(.) is a *response function*, which to a degree determines the distribution of the response. Since *F*(.) is increasing for fixed threshold *y*, the probability of a response larger than *y* increases with increasing person parameter $\theta _p$. Thus, $\theta _p$ can be seen as an ability or attitude parameter, which indicates the tendency of a person to obtain a high score. Higher values of $\theta _p$ are associated with a greater chance of a correct or affirmative response to each item. The name of the model refers to the modeling of the threshold *y*. It is not a threshold on the latent scale, which are the thresholds that are usually considered in latent trait modeling but on the *observable* scale.

In addition to the link between the latent variables and the observable response specified in equation (1), conditional independence of observable variables given the latent variables is assumed, which is a typical assumption in item response theory often referred to as local independence, e.g., Lord (1980). Conditional independence together with the latent monotonicity makes the model a monotone latent variable model in the sense of Holland and Rosenbaum (1986). Latent monotonicity as defined by Holland and Rosenbaum (1986) means that the probability $P(Y_{pi} > y|\theta _p,\delta _{i}(.))$ is a nondecreasing function of the person parameter for all items. Since the response function *F*(.) and the item difficulty functions are monotone, latent monotonicity holds for the thresholds model.

The specifics of the thresholds model follow from the functions that are chosen. The item difficulty function $\delta _{i}(.)$ contains the properties of the item, in particular if it is easy or hard to score high. It also determines the concrete form of the response distribution, which is only partially determined by *F*(.). In the following, it is shown that the model allows for quite different distributions of responses although the function *F*(.) is chosen fixed.

Latent trait models have been extensively discussed for binary or other categorical responses. Nevertheless, we start with the less familiar case of continuous responses and first investigate the potential of the thresholds model as a latent trait model for continuous responses.

### Linear Item Difficulty Functions

A particular interesting item difficulty function is the linear one, which allows for some simplifications. Let $Y_{pi}$ be a continuous response variable and the item difficulty be linear, $\delta _{i}(y)= \delta _{0i}+ \delta _i y$, $\delta _i > 0$. Then, one has a parametric model with item parameters $\delta _{0i},\delta _i$. One obtains that the expectation and variance of $Y_{pi}$ are given by

2 $$\begin{aligned} {\text {E}}(Y_{pi}) = \gamma _i\theta _p - \gamma _{0i},\quad {\text {var}}(Y_{pi}) = c \gamma _i^2/\alpha _i^2, \end{aligned}$$

where $\gamma _i=1/\delta _i$, $\gamma _{0i} =(\delta _{0i}+d/\alpha _i)/\delta _i$, with constants *d*, *c* that are determined by the distribution function *F*(.), for the concise form of constants and a proof, see “Appendix”. In addition, for symmetric response function *F*(.) the distribution function of $Y_{pi}$ is a shifted and scaled version of *F*(.).

It is immediately seen that high ability $\theta _p$ indicates a tendency to high responses. The item parameter $\gamma _i$ is a scaling parameter, and $\gamma _{0i}$ is the location on the latent scale. It represents the ’basic’ difficulty of the item; if $\gamma _{0i}$ is large, the expected response is small, and vice versa. The specific choice $\gamma _i=1$ (equivalent to $\delta _i=1$) and $\alpha _i=1$ yields the simpler forms ${\text {E}}(Y_{pi}) = \theta _p - \gamma _{0i}, {\text {var}}(Y_{pi}) = c$, which means that the response is simply determined by the difference between ability $\theta _p$ and item difficulty $\gamma _{0i}$, a property that is familiar from the binary Rasch model or the normal-ogive model without a slope parameter. If *F*(.) is symmetric, $d=0$, which means the expectation does not depend on the discrimination parameter. It, however, determines the variance such that large values of the discrimination parameter are associated with small variances of the response.

### The Person Threshold and the Item Characteristic Function

The link between the person and the difficulty functions can be described and visualized in several ways. An important function is the *person threshold function* (PT function), which for fixed $\theta _p$ is defined by

$$\begin{aligned} g_{i,\theta _p} (y) =P(Y_{pi} > y|\theta _p,\alpha _i,\delta _{i}(.)) = F(\alpha _i(\theta _p-\delta _{i}(y))), \end{aligned}$$

It shows the probability of an response above *y* for a specific person with ability $\theta _p$. It is strongly related to the distribution function of $Y_{pi}$, which is simply given by $F_{{pi}}(y) = 1-F(\alpha _i(\theta _p-\delta _{i}(y)))$. The distribution function is denoted with the subscript *pi* to distinguish it from the response function *F*(.) (which itself is a distribution function).

The second function is the general *item characteristic function* (IC function). It is an extended form of the item characteristic function commonly used in binary item response theory, and is defined by

$$\begin{aligned} IC_{i,y} (\theta _p) =P(Y_{pi} > y|\theta _p,\alpha _i,\delta _{i}(.)) = F(\alpha _i(\theta _p-\delta _{i}(y))). \end{aligned}$$

It shows the probability for an response above a fixed value *y* for varying abilities. In contrast to binary models where only the value $y=0$ is interesting, for responses with more than two possible values one has more than one function. If the response is continuous any value *y* can occur. Thus, the functions depend on the item *i* *and* *y*.

**Fig. 1:**
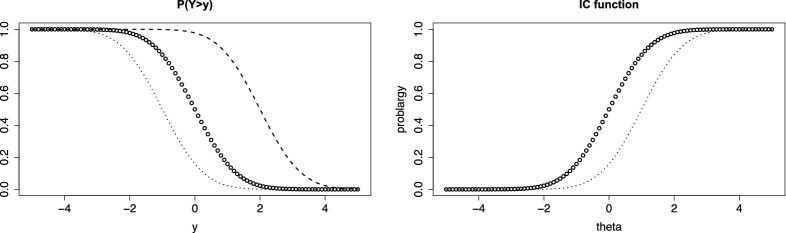
Left: Person threshold functions, $P(Y>y)$, for values $\theta =0$ (circles), $\theta =2$ (dashed), $\theta =-1$ (dotted); right: item characteristic functions for $y=0$ (circles) and $y=1$ (dotted)

For illustration we first consider the simple case of linear difficulty functions ($\alpha _i=1$). The left picture in Fig. 1 shows the person threshold function for three values of $\theta$ if *F*(.) is the normal distribution function and the threshold function is linear, $\delta (y)=y$. It is seen that a person with $\theta =2$ (dashed lines) has higher probability of a response above *y* than a person with $\theta _p=0$ (circles) for all values *y*. The right picture shows the IC function for two values of *y*, $y=0$ (circles) and $y=1$ (dotted). It is seen that the probability of a response above *y* is strictly increasing with ability $\theta$. In this simple case, the IC functions for different *y* are just shifted versions of the same basic normal distribution function. This changes with the parameters of the item difficulty function.

**Fig. 2:**
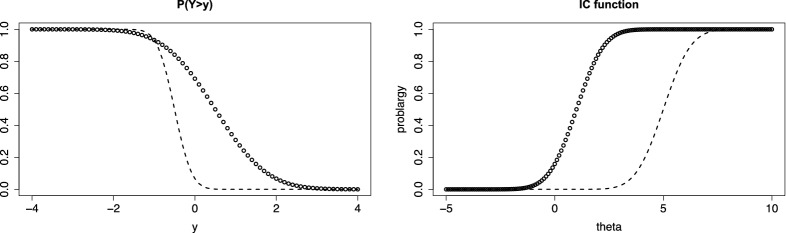
Left: Person threshold functions, $P(Y>y)$, for value $\theta = 0.5$ and $\delta _{i}(y)=y$ (circles), $\delta _{i}(y)=2+3y$ (dashed); right: item characteristic functions for the two items for $y=1$

Therefore, let us consider the parameters of the difficulty function in more detail. The first parameter $\delta _{0i}$ in the difficulty function $\delta _{i}(y)= \delta _{0i}+ \delta _i y$ determines the location of the item. The corresponding mean of $Y_{pi}$ is $-\delta _{0i}/\delta _i$ (for $\theta _p=0$ and symmetric function *F*(.)). Thus, the PT function is shifted to the left for large location parameter $\delta _{0i} >0$, which represents the basic difficulty. The second parameter determines the variance of $Y_{pi}$, large $\delta _i$ means that the variance is small. The left picture in Fig. 2 shows the PT functions for the simple function $\delta _{i}(y)=y$ (circles) and $\delta _{i}(y)=2+3y$ (dashed). It is seen that for the latter difficulty function the PT function is shifted to the left and the variance is much smaller, which is seen from the steep decrease of the dashed function. The right picture shows the corresponding item characteristic functions.

If the item discrimination parameter is the same for all items, the IC functions have the same form for all items, namely that of the distribution function *F*(.). This is immediately seen from the definition of the function $IC_{i,y} (\theta _p) = F(\alpha _i(\theta _p-\delta _{i}(y)))$ since for fixed value *y* the value of $\delta _{i}(y)$ is fixed. It is an important aspect regarding interpretation. For any *y* the item characteristic functions are increasing, and never do cross. That means a person with larger $\theta _p$ than another person has always a larger probability of a response above threshold *y*. This property, which is well known from binary Rasch models, also holds for the continuous thresholds model with fixed $\alpha _i$. It holds in spite of the scaling of the person parameter in the term $\gamma _i\theta _p$ of Eq. (2). If the binary Rasch model is extended to the 2PL model, often referred to as Birnbaum or 2PL model, item characteristic functions typically cross, as they do in the general thresholds model with a discrimination parameter.

The simplicity of the IC functions for fixed discrimination parameters has an additional advantage. Since all IC functions are just shifted (and possibly scaled) versions of the same function, they show which items are harder, and which are easier to solve. One obtains an ordering without having to investigate the item parameters.

The essential properties of the model, which hold for all sorts of responses to be considered later, can be described by the following functions, which refer to different aspects of the model.

- The item difficulty function, which characterizes the difficulty of the item over the whole range of possible outcomes.
- The person threshold function, which represents the distribution of the responses. The concrete form of the distribution as well as the support (see below) depend on the difficulty functions. The distributions may take quite different forms for different difficulty functions.
- The form of the item response function is kept fixed, for all items the probability of scoring above the threshold *y* increases in the same way with the ability. However, it depends on the threshold *y*, respectively, the corresponding $\delta _{i}(y)$, how large the probability of an response above *y* is.

It is essential to distinguish between two specifications regarding the complexity of the difficulty function. Let, more general, the difficulty functions be given by $\delta _{i}(y)= \delta _{0i}+ \delta _i g(y)$, where *g*(.) is a monotonically increasing function. Then, a simplifying assumption is that the difficulty functions have common slopes, that is, $\delta _1=\dots =\delta _I=\delta$. Without this restriction slopes may vary across items. For linear difficulty functions and $\alpha _i=1$ the assumption of common slopes simply means that for all responses one assumes the same variance.

If difficulty functions have the form $\delta _{i}(y)= \delta _{0i}+ \delta _i g(y)$ with fixed function *g*(.), the model is parametric with $\alpha _i,\delta _{0i},\delta _i$, $i=1,\dots ,I$, representing the item parameters, and $\theta _p$, $p=1,\dots ,P$, representing the person parameters. To obtain identifiability, some restrictions are needed. One can, for example, choose fixed values for one discrimination parameter and one person parameter (e.g. $\alpha _1=1,\theta _1=0$). In general, restrictions can depend on the support of the response, see Proposition 8.2.

Difficulty functions of the form $\delta _{i}(y)= \delta _{0i}+ \delta _i g(y)$ contain the slopes $\delta _i$. The concept of slopes should be distinguished from the concept that is sometimes used in binary and polytomous models. In binary models as the 2PL model, $P(Y_{pi}=1)=F(\alpha _i(\theta _p-\delta _{i}))$, the parameter $\alpha _i$ is a discrimination parameter, but is also often referred to as slope parameter. It has a quite different meaning than the slope in thresholds models. Large discrimination parameters have the effect that the increase in probability is stronger when $\theta _p$ increases than for smaller discrimination parameters. For fixed $\theta _p,\delta _{i}$ larger values of $\alpha _i$ mean that response probabilities are more extreme than for smaller values (closer to 1 if $\theta _p-\delta _{i}>0$, closer to 0 if $\theta _p-\delta _{i}<0$). The slopes in the difficulty functions of thresholds models have a different effect, they refer to the difficulty of items. As seen from equation (2) in the case of linear difficulty functions if the slope $\delta _i$ increases the expectation of responses decreases. Larger slopes means smaller expected responses, although also the variance changes. To keep the two concepts apart, we always refer to $\alpha _i$ as a discrimination parameter, and the notion of “slope” refers to the slope of the difficulty function.

As in other IRT models, alternative parameterizations can be used. The predictor $\eta _{pi}=\alpha _i(\theta _p-\delta _{0i}- \delta _i g(y))$ can also be given in the form $\eta _{pi}=\alpha _i\theta _p-\tilde{\delta }_{0i}- \tilde{\delta }_i g(y)$, where $\tilde{\delta }_{0i}=\alpha _i\delta _{0i}$, $\tilde{\delta }_i=\alpha _i\delta _i$. In the alternative parameterization, only the first term contains the person parameter, the intercept and slope are built from the item discrimination parameter and the original intercept and slope. The parameterization is helpful to clarify the role of slopes and discrimination parameters. The expectation of the response given in equation (2) suggests that the item slope (of the original parameterization) is the essential scaling parameter that acts as a discrimination parameter or factor loading, and $\alpha _i$ seems superfluous. But for linear difficulty functions (and symmetric response function) one obtains in the alternative parameterization ${\text {E}}(Y_{pi}) = (\alpha _i\theta _p - \tilde{\delta }_{0i})/\tilde{\delta }_i$, which shows that $\theta _p$ is weighted by $\alpha _i/\tilde{\delta }_i$. Thus, the expectation is determined by $\alpha _i$ and $\tilde{\delta }_i$ if one uses the alternative parameterization. The original parameterization $\alpha _i,\delta _{0i},\delta _i$ has the advantage that it is closer to parameterizations that are typically used in traditional binary and multi-categorical models. The alternative parameterization is useful in extensions to multi-dimensional structures to be considered later.

### Links to Classical Test Theory

The thresholds model for continuous responses is a genuine latent trait model. It has all the attributes of a latent trait model, items are located on the same scale as the latent ability, the latent variable accounts for observed interrelationship among the item responses and responses are described by a probabilistic model to explain their distribution. In contrast, classical test theory, which is often used for continuous data, is not a latent trait model in this sense. It is a regression type model, in which an a priori score on the entire test is chosen by assuming an additive decomposition of an observed test score into a true score and a random error; it can be traced back to Spearman (1904), an extensive presentation is found in Lord and Novick (1968).

A similar decomposition is obtained for the thresholds model with linear difficulty functions. If it holds one has

$$\begin{aligned} Y_{pi} = E(Y_{pi}) + E_{pi}, \end{aligned}$$

where ${\text {E}}(Y_{pi}) = \gamma _i\theta _p - \gamma _{0i}$, ${\text {var}}(E_{pi}) = c \gamma _i^2/\alpha _i^2$ and $Y_{pi}$ has distribution function *F*(.). Random sampling of individuals yields

$$\begin{aligned} Y_{*i} = E(Y_{*i}) + E_{*i}, \end{aligned}$$

which corresponds to the decomposition into a true-score and an error-score random variable (see Section 2.6 Lord and Novick, 1968) with the true score depending only on the measurement instrument. The error-score $E_{pi}$ follows distribution function *F*(.), and has expectation 0 and variance $c\gamma _{i}^2/\alpha _i^2$. In classical test theory, the variance of the error-score is often assumed to be the same for all responses, which means that in addition $\gamma _i =\gamma ,\alpha _i=\alpha$ holds for all *i*.

Models for continuous responses have also been considered by Mellenbergh (2016) including Spearman’s one factor model and the model for congeneric measurements and Noel and Dauvier (2007), who proposed a beta item response model. The models considered there are, as the classical test theory, rather restrictive since the response is assumed to be a linear function of the latent traits.

### Alternative Item Difficulty Functions

If difficulty functions are linear, the responses follow the distribution function *F*(.). However, responses come with quite different distributions. They can be strictly positive, for example if the response time is an indicator of the ability of a person, or they are restricted to specific intervals, for example if a person scores in a given interval continuously or approximately continuously by using numbers, say $1, 2,\dots , 100$. In both cases a normal distribution is inadequate, although in the latter case with numbers $1, 2,\dots , 100$ investigators typically use a normal distribution in spite of the problems that occur at the boundaries of the interval.

**Fig. 3:**
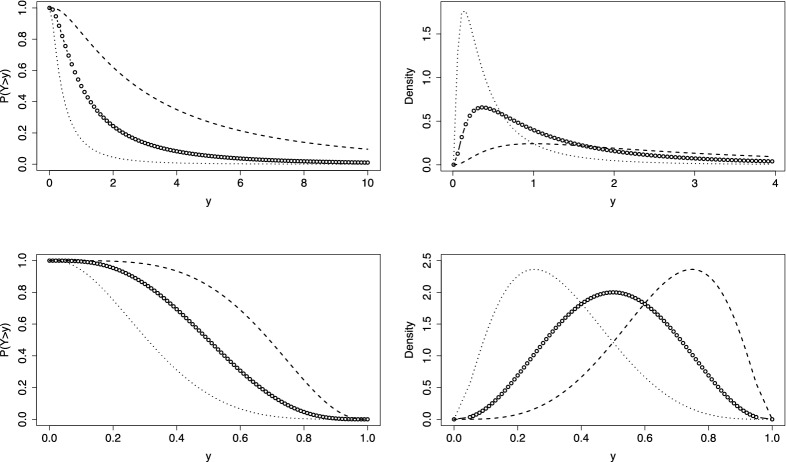
In the first row the item difficulty is $\delta (y)=\log (y)$ (for non-negative responses), in the second row the item difficulty is $\delta (y)= F^{-1}(y)$ ( responses in [0, 1]). Left column shows $P(Y>y)$ for values $\theta =0$ (circles), $\theta =1$ (dashed), $\theta =-1$ (dotted); right column shows the corresponding densities

A strength of the thresholds model is that it allows to account for the support of the response by using specific difficulty functions. Let the response function *F*(.) again be the standard normal distribution and the item difficulty be given by $\delta (y)=\log (y)$. Figure 3 (left, first row) shows the person threshold functions ($\alpha =1$) for persons with parameters $\theta =0$ (circles), $\theta =1$ (dashed) and $\theta =-1$ (dotted). Although a normal distribution is assumed for the response function, the response is strictly positive, and definitely not normally distributed, as is seen from the corresponding densities (right, first row).

In practice, test data are always restricted to specific finite values. A prominent case is Likert-type responses on 5 or 7-point scales. Although values are definitely discrete, often they are considered as continuous and common distributions as the normal distribution are assumed. The problem that responses at the boundary cannot follow a normal distribution is typically ignored. For truly continuous response, scales represented by continuous line segments Samejima (1973) extended the graded response model to responses to an open line segment, and Müller (1987) extended the rating scale model to responses to a closed line segment. Both extensions are derived as limiting cases of discrete response models.

The thresholds model offers an alternative way to account for the fact that data are restricted to a fixed interval, and specify a proper distribution for which the support is the interval in which responses are observed. Without loss of generality, one can choose the interval [0, 1] because data can always be transformed into that interval. Then, an attractive difficulty function that can be used is the inverse function $\delta (y)=aF^{-1}(y)$ with some constant a. The second row of Fig. 3 shows the person threshold functions and the corresponding densities if the response is restricted to the interval [0, 1], and $a=1$. It is seen that densities have support [0, 1] and are not normally distributed although the normal response function *F*(.) generates the distribution. For large $\theta _p$ the distribution is shifted to the right, but still within support [0, 1]. There is no mis-specification of the distribution for very large or small values of $\theta _p$, as occurs if one assumes a normal distribution for the response itself (instead of using a normal response function and appropriate difficulty functions in the thresholds model).

### Illustrative Application: Cognition Data

For illustration, we use the data set *Lakes* from the R package *MPsychoR* (Mair, 2018). It is a multi-facet G-theory application taken from Lakes and Hoyt (2009). The authors used the response to assess children’s self-regulation in response to a physically challenging situation. The scale consists of three domains, cognitive, affective/motivational, and physical. We use the cognitive domain only. Each of the 194 children was rated on six items on his/her self-regulatory ability with ratings being on a scale from 1 to 7. Mair (2018) used the data to illustrate concepts of classical test theory implicitly assuming a metric scale level.

**Fig. 4:**
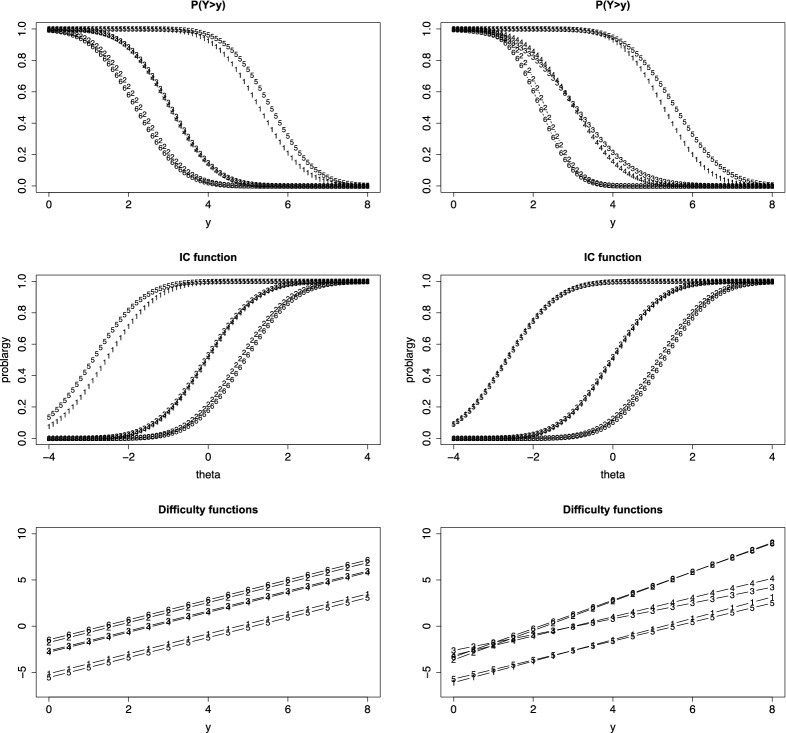
First row: person threshold functions, $P(Y>1)$, for cognition data ($\theta _p = 0$) and linear difficulty functions; second row: IC functions for $y=3$; left: common slopes are assumed, right: varying slopes; third row: difficulty functions

We fit a thresholds model with linear difficulty functions, normal response function and fixed discrimination parameters ($\alpha _i=1$). The first row of Fig. 4 shows the person threshold functions for $\theta _p = 0$, under the assumption of common slopes in the difficulty functions (left) and with possibly varying slopes (right). The numbers in the curves denote the items. It is seen that items 3 and 4 are hardly distinguishable, items 2 and 6 are harder and items 1 and 5 easier. It is seen from the right picture (varying slopes) that the variance of responses is smaller for items 2 and 6 when compared to the other items, which corresponds to the large estimated slopes of items 2 and 6 in Table 1. The second row of Fig. 4 shows the corresponding IC functions. It is seen that the distance between the pairs of items $\{3,4\}$ and items $\{2,6\}$ is larger if the model allows for varying slopes. The last row shows the difficulty functions. They are strictly parallel in the case of a common slope. For varying slopes the pairs of items are still close to each other but the items 2 and 6 have larger slopes. Table 1 shows the estimated parameters, standard errors for the intercept were between 0.152 (item 3) and 0.329 (item 1), for the slope between 0.043 (item 3) and 0.052 (item 5) in the model with varying slopes.

Since one has nested models, it is of interest if the model with varying slopes can be simplified to the model with common slopes in the difficulty functions. The corresponding log-likelihood test is 108.34 on 5 df, which clearly indicates that the simplified model is not adequate. As mentioned before, testing that slopes are constant means testing if the variances of the error score are constant, which seems not to be the case. We focused on the model with fixed discrimination parameters since varying discrimination parameters did not improve the fit. More concise, the log-likelihood for the model with varying discrimination was -1445.832, which is the same value as for the model with fixed discrimination parameter. The AIC for the latter is 2917.664, for the model with varying discrimination parameter one obtains 2927.664, which clearly favors the model with fixed discrimination parameters. With the exception of item 2, for all items the parameter estimates for the more general estimates were the same as for the model with fixed discrimination parameter. For item 2 the estimates of the intercept and slope were -2.236756 and 0.9694296, and the estimate of the discrimination parameter was 1.630, for all other items the estimate was 1.

**Table 1:** Estimated parameters for cognition data

Item	Common slope		Varying slopes	
	Intercept	Slope	Intercept	Slope
1	$-$ 5.935405	1.123857	$-$ 6.302662	1.1934046
2	$-$ 2.592028	1.123857	$-$ 3.646508	1.5804284
3	$-$ 3.452920	1.123857	$-$ 2.635715	0.8577245
4	$-$ 3.407838	1.123857	$-$ 3.149895	1.0391432
5	$-$ 6.269576	1.123857	$-$ 5.747873	1.0304311
6	$-$ 2.451351	1.123857	$-$ 3.367810	1.5432459
Log-lik	$-$ 1500.006		$-$ 1445.832	

## Discrete Responses

In the following, first it is shown that classical models for binary and ordered responses can be represented as thresholds models. Then, models with infinite support are considered.

### Binary and Ordered Categorical Responses

Let us start with the simplest case of a binary response variable $Y_{pi} \in \{0,1\}$. Then, the only relevant value of the function $\delta _{i}(y)$ is $\delta _{i}(0)=\delta _{0i}$ because $P(Y_{pi} > 0) = P(Y_{pi} =1)$, and if $P(Y_{pi} =1)$ is known, all response probabilities are known. The thresholds model yields immediately the binary response model

$$\begin{aligned} P(Y_{pi} =1|\theta _p,\alpha _i,\delta _{i}(.)) = F( \alpha _i(\theta _p - \delta _{0i}) ). \end{aligned}$$

Thus, if *F*(.) is chosen as normal distribution one obtains the normal-ogive model, if *F*(.) is the logistic distribution function one obtains the 2PL model, which simplifies to the binary Rasch model if $\alpha _i=1$ (Rasch, 1961).

The binary case makes it clear why the difficulty function is defined on the support *S* of $Y_{pi}$ rather than on the whole field of real numbers. For $Y_{pi} \in \{0,1\}$ one has to consider only $\delta _{i}(0)$ and $\delta _{i}(1)$. For the latter one has $\delta _{i}(1)=\infty$ since $P(Y_{pi} >1)=0$. For the general case see Proposition 8.2 in the “Appendix”.

Let now $Y_{pi} \in \{0,\dots , k\}$ be a response variable with ordered categories, and let the difficulty functions $\delta _i(y)$ be restricted only by the assumption that it is a strictly monotonically increasing function. Let parameters be defined by $\delta _{ir}=\delta _i(r-1)$. Then, one obtains the thresholds model

$$\begin{aligned} P(Y_{pi} \ge r|\theta _p,\alpha _i,\delta _{i}(.)) = F( \alpha _i(\theta _p - \delta _{ir}) ), \quad r=1,\dots , k, \end{aligned}$$

which is a well-known model, namely Samejima’s graded response model (Samejima, 1995, 2016).

To obtain the graded response model without further constraints, it is essential that the form of the difficulty functions is restricted by the monotonicity assumption only. The monotonicity assumption itself is indispensable because otherwise the thresholds model would not be defined. Nevertheless, it is again interesting to consider the model with a pre-specified threshold function. If $\delta _{i}(y)= \delta _{0i}+ \delta _i y$ holds, one obtains that differences between adjacent item parameters are constant, $\delta _{ir}-\delta _{i,r-1}=\bar{\delta }_i$. In this simplified version of the graded response model, each item is characterized by just three parameters, the item discrimination, the location $\delta _{0i}$ and the slope $\delta _i$. It reduces the number of parameters in a similar way as the Rasch rating scale model (Andrich, 1978, 2016) reduces the number of parameters in the partial credit model. Simplified versions also result from using alternative *fixed* difficulty functions, for example, the log function $\delta _{i}(y)= \delta _{0i}+ \delta _i \log (y)$, which has been used above to obtain $Y_{pi} \ge 0$, or the inverse function, which can been used to restrict responses to fixed intervals.

In particular, if the number of categories is large or medium sized, as for example in a 9-point rating scale, it is tempting to assume that responses are (approximately) continuous and use corresponding modeling approaches, a strategy that is often found in applied research, see also Robitzsch (2020). The graded response model takes the support seriously, it is a model that explicitly assumes that the response is discrete and therefore follows a multinomial distribution. The thresholds model, which contains the graded response model as a special case, is quite flexible concerning the assumption of the support. In the general model formulation, $P(Y_{pi} > y|\theta _p,\alpha _i,\delta _{i}(.))=F( \alpha _i(\theta _p-\delta _{i}(y)) )$, only the effect of the ability and the item difficulty function on the probability of a response above threshold *y* is fixed. It applies to continuous as well as discrete data. Of course, when estimating by maximum likelihood methods, one has to distinguish between the discrete and the continuous case since the densities have to be specified. However, in practice the estimated difficulty functions are very similar (see next section), the crucial part is indeed the specification of the response function *F*(.) and the difficulty function.

The thresholds model can be seen as bridging the gap between continuous and discrete responses. The bridging can be made more explicit in the case of the graded response function. As shown in the “Appendix”, there is a strong link between the continuous thresholds model and the graded response model since the thresholds model $P(Y_{pi} > y)=F( \alpha _i(\theta _p-\delta _{i}(y)) )$ holds for continuous response $Y_{pi}$ if and only if the graded response model holds for all categorizations

$$\begin{aligned} Y_{pi}^{(c)} =r \quad \Longleftrightarrow \quad Y_{pi} \in (\tau _{r}, \tau _{r+1}], \end{aligned}$$

where $\tau _{1}< \dots < \tau _{k}$ are any ordered thresholds. Since the graded response model itself is a thresholds model, this means that thresholds models are stable under categorization, that is, they also hold if one considers categorized versions of the response. It should be noted that observable responses are considered, the result differs from the usual result that the graded response is a categorized version of a *latent* variable.

### Political Fears

As an illustrating example, we consider data from the German Longitudinal Election Study (GLES), which is a long-term study of the German electoral process (Rattinger et al., 2014). The data we are using originate from the pre-election survey for the German federal election in 2017 and are with political fears. The participants were asked: “How afraid are you due to the ...”—(1) refugee crisis?—(2) global climate change?—(3) international terrorism?—(4) globalization?—(5) use of nuclear energy? The answers were measured on Likert scales from 1 (not afraid at all) to 7 (very afraid). The model is fitted under the assumption that fear is the dominating latent trait, which is considered as unidimensional. We use 200 persons sampled randomly from the available set of observations.

**Fig. 5:**
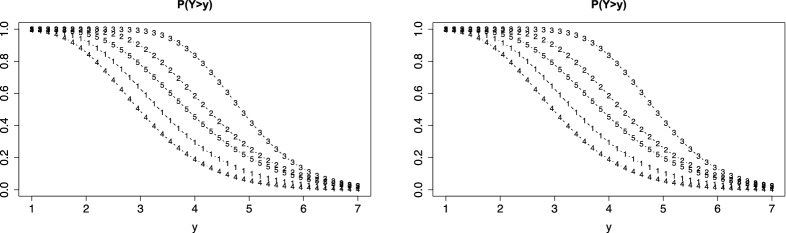
PT functions for political fear data with logarithmic difficulty functions, varying slopes and discrimination parameters, left: discrete distribution, right: continuous distribution

Figure 5 shows the person threshold functions obtained when using logarithmic difficulty functions, varying slopes and discrimination parameters. The left picture shows the fitted functions when assuming a discrete, multinomial distribution, the right picture when assuming a continuous distribution. It is seen that the fitted person threshold functions are rather similar. In both cases, varying slopes are needed (likelihood ratio test yields 39.66 for discrete distribution, 32.14 for continuous distribution on 4 df). Also varying discrimination parameters seem more appropriate (likelihood ratio test 8.844 for continuous distribution, 9.536 for discrete distribution on 4 df). Table 2 shows the estimates for the model with varying slopes and fixed discrimination parameters and the model with varying discrimination parameters.

**Table 2:** Estimated parameters for fears data

Item	Fixed discrimination			Varying discrimination		
	Intercept	Slope	$\alpha _i$	Intercept	Slope	$\alpha _i$
1	$-$ 4.840	3.056	1	$-$ 4.353	2.749	1.086
2	$-$ 6.661	3.807	1	$-$ 5.399	3.086	1.225
3	$-$ 7.569	4.093	1	$-$ 4.730	2.557	1.868
4	$-$ 4.571	3.040	1	$-$ 3.661	2.434	1.241
5	$-$ 5.716	3.400	1	$-$ 5.424	3.226	1.000
Log-lik	$-$ 1979.395			$-$ 1974.973		

### Discrete with Infinite Support: Count Data

Measurement of cognitive abilities often uses count data, for example, the number of remembered stimuli (Süß et al., 2002), or the number of generated ideas in a fixed time interval (Forthmann et al., 2017), for an overview see also Forthmann et al. (2020). In all these cases, the responses are counts with $Y_{pi} \in \{0,1,2,\dots \}$. A classical model that has been used for this kind of data is Rasch’s Poisson count model Rasch (1960), which has been extended to the Conway–Maxwell–Poisson model by Forthmann et al. (2020).

**Fig. 6:**
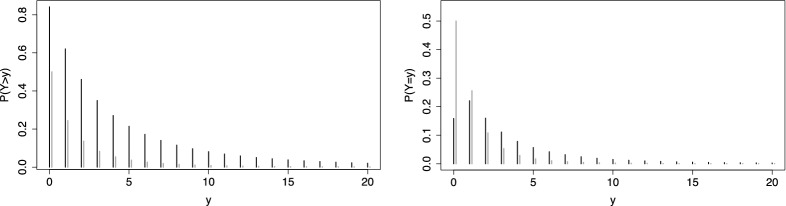
Left: $P(Y>y)$ for values $\theta =1$ (bold), $\theta =0$ (gray) for count data with item difficulty function $\delta _i(y) = \log (y+1)$; on the right-hand side the corresponding probability mass functions are shown

The thresholds model is a flexible alternative to these models. An attractive choice of a fixed difficulty function is the $\log$-function in the form $\delta _i(y) = \log (y+1)$. Figure 6 shows the person threshold functions and the densities for two values of person parameters, $\theta =1$ (bold) and $\theta =0$ (gray), where *F*(.) is the standard normal distribution function. It is seen that the PT function for $\theta =1$ is always larger than the PT function for $\theta =0$ . The densities show that the persons with $\theta =1$ tend to score higher than persons with $\theta =0$. The IC functions are not shown since by construction they have the form of a normal distribution.

The flexibility of the count thresholds model is comparable to the Conway–Maxwell–Poisson model if the difficulty functions are specified by $\delta _i(y) =\delta _{0i} + \delta _i\log (y+1)$ since the slope $\delta _i$ allows for additional variability of the response across items.

### Verbal Fluency Data

Forthmann et al. (2020) used a data set with four commonly used verbal fluency tasks, which they were so kind to let us use for illustration. The data set includes two semantic fluency tasks, namely animal naming (item 1) and naming things that can be found in a supermarket (item 4) and two letter fluency tasks, words beginning with letter f (item 2) or letter s (item 3). The 202 participants had one minute to complete each of the verbal fluency tasks.

**Fig. 7:**
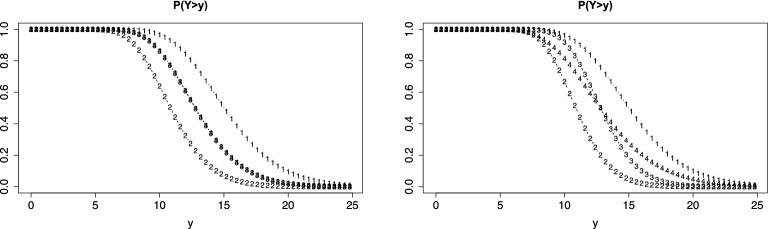
Person threshold functions, $P(Y>y)$, for value $\theta = 0$ and $\delta _{i}(y)=\delta _{0i}+\delta _{i}\log (1+y)$ for verbal fluency data assuming a discrete distribution; left: common slope, right: varying slopes

Figure 7 shows the person threshold functions for common slope (left) and for varying slope (right) if count data are considered as discrete (loglik = -2065.42, $\sigma _{\theta }= 1.04$ for common slope, loglik = -2038.58, $\sigma _{\theta }= 1.09$ for varying slopes). It is seen that under the assumption of a common slope items 3 and 4 have virtually the same threshold function, item 1 allows for higher responses, item 2 is harder, and counts tend to be lower. If slopes are allowed to vary over items, the order of items remains the same, but items 3 and 4 have slightly different functions. Item 3 shows a more distinct decrease indicating smaller dispersion than item 4. We also fitted the model with varying discrimination parameters, but there is no indication that they are needed (loglik = -2036.004, $\sigma _{\theta }= 0.92$).

The functions given in Fig. 7 are obtained by explicitly using the support $\{0,1,2,\dots \}$, and therefore assuming a discrete distribution. Thus, the curves should be interpreted only at values $\{0,1,2,\dots \}$, only for simplicity of presentation they were shown as continuous functions.

Since counts are on a metrical scale one could also think of fitting a model that assumes a continuous response, and consider it as an approximation. We fitted the corresponding thresholds model and obtained virtually the same functions as given in Fig. 7, which are therefore not shown. Of course the likelihood values differ from the values obtained by using a discrete model. However, inference yields similar results. The likelihood ratio test that compares the model with common slopes in the difficulty functions to the model with varying slopes is 53.68 for the discrete model and 54.08 for the continuous model (on 3 df). Thus, in both cases the model with varying slopes turns out to be more appropriate.

The thresholds model is an alternative to more classical approaches to model count data as Rasch’s Poisson count model (Rasch, 1960). In additive parameterization, the model specifies the expected response $\mu _{pi}$ for person *p* on item *i* by $\mu _{pi} = \exp (\theta _p - \delta _i)$, where $\theta _p$ is the person ability and $\delta _i$ the item difficulty. For the distribution, a Poisson distribution is assumed. Fitting of the model yields the log-likelihood -2072.40 and for AIC 4154.58. Comparison with the thresholds model with varying slopes (log-likelihood -2038.06 and AIC 4094.11) shows that the thresholds model shows superior fit.

## Further Properties and Alternative Modeling Approaches

### Choice of Difficulty Functions

Item responses can be continuous or discrete, in the latter case the number of categories can be finite or infinite. As has been illustrated in the previous sections, in particular the difficulty function determines the range of the response. It therefore has to be adapted to the item type. Table 3 gives an overview of possible combinations of item types and difficulty functions, where it is assumed that the response function *F*(.) is symmetric and continuous with support $\mathbb {R}$.

If there is reason to assume that responses follow a specific continuous distribution function $F_{\text {resp}}(.)$, for example the normal distribution, the thresholds model with response function $F(y)=F_{\text {resp}}(y)$ and linear difficulty functions is a natural choice. Then, the distribution of $Y_{pi}$ has distribution function $F_{\text {resp}}(.)$ with the mean and the variance determined in a simple way (see Sect. 1.1). For the density, one obtains the simple form $f((y-\mu _{pi})/\sigma _{pi})/\sigma _{pi}$, where $f(.)=F'(.)$ is the density of the response, and $\mu _{pi}$, $\sigma _{pi}^2$ are the mean and variance of $Y_{pi}$ (Proposition 8.1 in “Appendix”). Although the form of the density reminds of the normal distribution, it holds for any symmetric distribution.

For items with responses $Y_{pi}>0$, difficulty functions should be chosen such that $\lim _{y\rightarrow 0}\delta _i(y)=-\infty$ to ensure that $Y_{pi}>0$ holds. As an example, the logarithmic function is given in Table 3. If responses are from a known interval (*a*, *b*), difficulty functions should fulfill $\lim _{y\rightarrow a}\delta _i(y)=-\infty$, $\lim _{y\rightarrow b}\delta _i(y)=\infty$. After a transformation of the responses into the interval (0, 1), natural choices are inverse distribution functions, for example, $g(y)=aF^{-1}(y)$ or the logit transformation $g(y)=\log (y/(1-y))$.

In general, for continuous responses the difficulties determine the distributions of the response. They do not necessarily follow classical response distributions. However, some classical distributions can be obtained by choosing specific difficulty functions. If one chooses the linear difficulty function, one obtains the distribution that is assumed for the response function. In particular, if the response function is the normal distribution, responses are normally distributed. A combination that also yields a classical distribution is the normal response function together with the logarithmic difficulty function. Then, one obtains for the responses the log-normal distribution with density $1/(y\sqrt{2\pi }\bar{\sigma }_i) \exp (-(\log (y)-\bar{\mu }_{pi})^2/(2\bar{\sigma }_i^2))$, where $\bar{\mu }_{pi}= (\theta _p-\delta _{0i})/\delta _i$, $\bar{\sigma }_i=1/(\alpha _i\delta _i)$. In other combinations of response and difficulty functions, one specifies the difficulty function instead of choosing a response distribution as in more traditional modeling approaches.

If responses are discrete and have finite support, the response distribution is always the multinomial distribution. The only values of the difficulty function that enter the model are $g(0),\dots , g(k-1)$ (for $Y_{pi} \in \{0,1,\dots ,k\}$). In the binary case, the choice of the difficulty function is irrelevant since $\delta _{0i}+ \delta _i g(0)$ can always be condensed into one intercept parameter $\tilde{\delta }_{0i}=\delta _{0i}+ \delta _i g(0)$. A simple function that has been used in applications is the logarithmic function $g(y)=\log (1+y)$, which is evaluated at $0,1,\dots , k-1$. However, alternative functions could be constructed. One alternative is the adapted logit function $g(y)=\log ((1+y)/(k-y))$. It is symmetric around $m=(k-1)/2$, such that $g(m+a)=-g(m-a)$ and is steeper than $\log (1+k)$ for large values of *y*. The symmetry makes it attractive since it entails more symmetric distributions, for example, one obtains $P(Y_{pi}=0)=P(Y_{pi}=k)$ for $\theta _p=\delta _{0i}$. It extends more naturally to values beyond the support. While for both functions $\lim _{y\rightarrow -1}g(y)=-\infty$ holds, at the right boundary one has $\lim _{y\rightarrow k}g(y)=\infty$ for the transformed logit function while $\log (1+k)$ is a finite value. For count data there is no right boundary and there is no need for symmetry, which means that symmetric functions have no advantage over the logarithmic function.

**Table 3:** Item types and difficulty functions

Item type	Support	Difficulty function, $\delta _{i}(y)= \delta _{0i}+ \delta _i g(y)$	
Continuous	$Y_{pi} \in \mathbb {R}$	Linear	$g(y)=y$
	$Y_{pi} \ge 0$	Logarithmic	$g(y)=\log (y)$
	$Y_{pi} \in (0,1)$	Inverse	$g(y)=aF^{-1}(y)$
Discrete	$Y_{pi} \in \{0,1,\dots \}$	Logarithmic	$g(y)=\log (1+y)$
	$Y_{pi} \in \{0,1,\dots ,k \}, k >1$	Logarithmic	$g(y)=\log (1+y)$
		Logit	$g(y)=\log ((1+y)/(k-y))$
	$Y_{pi} \in \{0,1\}$		*g*(*y*) not used

### Item Information

In traditional IRT models, item information is considered a useful concept. In general item information (or Fisher information) for item *i* can be defined as the expectation

$$\begin{aligned} I(\theta _p)= {\text {E}}\left( - \frac{\partial ^2 l_i(Y;\theta _p)}{ \partial \theta _p \partial \theta _p}\right) , \end{aligned}$$

where $l_i(Y;\theta _p)$ is the log-likelihood for item *i* and observation *Y*. For discrete $Y \in \{0,1,\dots \}$, the log-likelihood is given by $l_i(Y;\theta _p)= \sum _r Y_r \log (\pi _{ir}(\theta _p))$, where $Y_r=1$ if $Y=r$, $Y_r=0$ otherwise, and $\pi _{ir}(\theta _p)=P(Y=r|\theta _p,\alpha _i,\delta _{i}(.))$ is the probability of a response in category *r* on item *i*. One obtains

$$\begin{aligned} I(\theta _p)= \sum _r \frac{\pi _{ir}'(\theta _p)^2-\pi _{ir}(\theta _p)\pi _{ir}''(\theta _p)}{ \pi _{ir}(\theta _p)}, \end{aligned}$$

where $\pi _{ir}'(\theta _p)$ and $\pi _{ir}''(\theta _p)$ are the first and second derivatives of $\pi _{ir}(\theta _p)$ with respect to $\theta _p$. For example, for the Rasch model one obtains the simple form $I(\theta _p)=\pi _{i0}(\theta _p)\pi _{i1}(\theta _p)=(1-\pi _{i1}(\theta _p))\pi _{i1}(\theta _p)$. The information in more general binary models has been considered, for example, by Lord (1980), Magis (2013).

If the number of categories is finite, $Y \in \{0,\dots ,k \}$, a simpler form can be derived, in which second derivatives are not needed. By using $\pi _{i0}(\theta _p)=1-\pi _{i1}(\theta _p)-\dots -\pi _{ik}(\theta _p)$ one obtains

$$\begin{aligned} I(\theta _p)= \sum _{r=1}^k \frac{\pi _{ir}'(\theta _p)^2}{\pi _{ir}(\theta _p)}+ \frac{(\pi _{i1}'(\theta _p)+\dots +\pi _{ik}'(\theta _p))^2}{1-\pi _{i1}(\theta _p)-\dots -\pi _{ik}(\theta _p)}. \end{aligned}$$

**Fig. 8:**
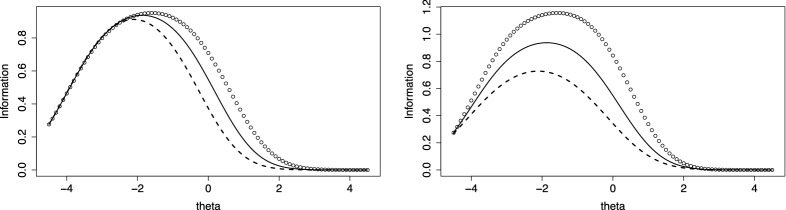
Information functions for three items with intercepts $\delta _{0i}=-3$, item 1: dotted line, item 2: drawn line, item 3: dashed line; left: varying item slopes, $(\delta _1,\delta _2,\delta _3)=(1.2,1.0,0.8)$, item discrimination fixed, $\alpha _i= 1$; right: varying item discrimination, $(\alpha _1,\alpha _2,\alpha _3)=(1.2,1.0,0.8)$

For illustration, Fig. 8 shows the information functions for three items with 10 response categories and logarithmic difficulty functions. Since varying intercepts yield just shifted versions of the information function we let all the intercepts be the same ($\delta _{0i}=-3$). The left picture shows the information functions for item slopes $(\delta _1,\delta _2,\delta _3)=(1.2,1.0,0.8)$ and $\alpha _i= 1$ for all items (item 1: dotted line, item 2: drawn line, item 3: dashed line). Since item one is the hardest its peak is at larger $\theta$-values than for the other two items. The right side shows the information function if in addition the $\alpha _i$s vary, $(\alpha _1,\alpha _2,\alpha _3)=(1.2,1.0,0.8)$. The discrimination parameter changes the range of the information function. Item 1, which has the largest discrimination parameter, yields the largest values. As has been shown for linear difficulty functions large values of $\alpha _i$ are linked to small variances of the response, an effect which is also present for nonlinear functions and explains why information is larger for large discrimination parameters.

For continuous responses, one obtains a closed form for the observed information only. It is given by $I_{\text {obs}}(\theta _p,Y)=-{\partial ^2 l_i(Y;\theta _p)}/{ \partial \theta _p \partial \theta _p}=\alpha _i^2((f'(\eta _{ipY})/f(\eta _{ipY}))^2-f''(\eta _{ipY})/f(\eta _{ipY}))$, where $\eta _{ipY}=\alpha _i(\theta _p-\delta _{i}(Y))$, *f*(.) is the derivative of *F*(.), and $f'(.)$, $f''(.)$ the first and second derivatives. The expected observation can be obtained by numerical integration. For normal distribution function *F*(.), it can be computed explicitly, yielding $I(\theta _p)=I_{\text {obs}}(\theta _p,Y)=\alpha _i^2$. Then, the information depends on the discrimination parameter only.

### Differential Item Functioning

Differential item functioning (DIF) is the well-known phenomenon that the probability of a correct response among equally able persons differs in subgroups. For example, the difficulty of an item may depend on the membership to a racial, ethnic or gender subgroup. Then, the performance of a group can be lower because these items are related to specific knowledge that is less present in this group. Various methods have been developed to avoid the potential measurement bias and discrimination, see, for example, Millsap and Everson (1993), Zumbo (1999), Rogers (2005), Osterlind and Everson (2009) and Magis et al. (2010).

For thresholds models, DIF can be investigated in a similar way as in approaches that have been used for the Rasch model, namely by including covariates in the model. Let $\varvec{x}_p$ a person-specific vector of covariates that contains, for example, gender, race, but also metric covariates like age. In a generalized thresholds model, the person parameter $\theta _p$ is replaced by $\theta _p+\varvec{x}_p^T\varvec{\gamma }_i$ yielding the differential item functioning thresholds model

$$\begin{aligned} P(Y_{pi} > y|\theta _p,\alpha _i,\delta _{i}(.))=F( \alpha _i(\theta _p+\varvec{x}_p^T\varvec{\gamma }_i-\delta _{i}(y)) ). \end{aligned}$$

The parameter $\varvec{\gamma }_i$ is item-specific and indicates the presence of DIF if it is unequal zero. The hypothesis $H_0: \varvec{\gamma }_i=\varvec{0}$ can be tested by using likelihood ratio test, which is easy to do in particular if one considers DIF in items one at a time. For Rasch models, which are special cases of the thresholds model, DIF detection of this type has been considered, for example, by Paek and Wilson (2011), however restricted to binary covariates, which distinguish between a focal and a reference group. If $\varvec{x}_p$ is vector-valued, and one wants to model DIF in all items simultaneously, simple marginal estimates will be hard to obtain for a larger number of items. More recently, penalty approaches have been proposed that also work for a larger number of items in Rasch models (Tutz and Schauberger, 2015). In penalty approaches, the likelihood is replaced by penalized likelihood with penalty terms that enforce selection of covariates. They work in a similar way as the penalty methods considered in Sect. 5 but with different penalty terms. The penalty terms used in these approaches can also be used in the estimation of thresholds models yielding a general concept of DIF modeling in thresholds models.

The differential item functioning thresholds model models DIF within a specific item response model as does the Rasch differential item functioning model considered by Paek and Wilson (2011). The approach has advantages over more traditional DIF detection approaches as Lord’s $\chi ^2$ test (Lord, 1980) and the logistic regression method (Swaminathan and Rogers, 1990; Magis et al., 2015). The latter approach uses the test score of respondents to act as matching variable and as a proxy for respondent’s ability. It assumes that test scores do represent the respondent’s ability, which might hold in special models but certainly not in general.

### Alternative Modeling Strategies

#### Nonparametric Item Response Models

A flexible class of models are nonparametric item response models (Mokken, 1971; Junker and Sijtsma, 2001; Sijtsma and Molenaar, 2016). The binary nonparametric homogeneity model assumes only local independence, unidimensionality, and monotonicity and therefore encompasses binary thresholds models. Thresholds models are more restrictive since they assume a fixed response function *F*(.), while in the homogeneity model the response functions can have any form provided they do not decrease. The flexibility of thresholds models refers to the distribution of the responses. In particular in models with more general difficulty functions to be considered later, the form of the distributions is hardly restricted. For binary responses, this flexibility is not exploited since the form of the response distribution is fixed to be a Bernoulli distribution.

One strength of the thresholds model is that it preserves the essential components of IRT models, but let distributions take various forms. It is able to not only fit binary responses but also count data and continuous responses, and, crucially, link it to the same latent construct. While nonparametric models provide maximal flexibility in characterizing the relationship between latent construct and item score, thresholds models do not aim at the item score, they aim at linking various possible distributions in a flexible way to person abilities.

#### Multidimensional IRT Models

Multidimensional IRT models provide an alternative extension of more classical response models, see, for example, Swaminathan and Rogers (2018); Chalmers (2012). The R package *mirt* (Chalmers, 2012) allows to fit binary and multi-categorical models as the graded response model with a multi-dimensional structure. The basic concept is to replace the unidimensional trait $\theta _p$ by an m-dimensional trait (latent factors) $\varvec{\theta }_p^T=(\theta _{p1},\dots ,\theta _{pm})$. Then, in the simple binary model one uses $P(Y_{pi}=1)=F(\alpha _{i0}+\varvec{\alpha }_i^T\varvec{\theta }_p)$, where $\alpha _{i0}$ is an item-specific intercept and $\varvec{\alpha }_i$ an item-specific parameter vector. In multi-categorical models, the intercepts are category-specific, and the model contains more than one threshold.

It is straightforward to use multi-dimensional structures in thresholds models. Instead of the predictor $\eta _{pi}=\alpha _i(\theta _p-\delta _{i0}- \delta _i g(y))=\alpha _i\theta _p- \tilde{\delta }_{i0}- \tilde{\delta }_i g(y)$, where $\tilde{\delta }_{i0}=\alpha _i\delta _{i0}$, $\tilde{\delta }_i=\alpha _i\delta _i$, one uses $\eta _{pi}=\varvec{\alpha }_i^T\varvec{\theta }_p- \tilde{\delta }_{i0}- \tilde{\delta }_i g(y)$. Then, if *g*(.) is kept flexible, for example by using basis functions, one obtains for categorical responses the multi-dimensional models used by Chalmers (2012). An advantage is that within this framework one also obtains multi-dimensional models for count data and continuous responses, and mixed item formats can be used within a test.

A caveat is that multi-dimensional models can yield paradoxical results (Jordan & Spiess, 2012), which might also be the case in multi-dimensional thresholds models. Nevertheless, specific multi-dimensional models turned out to be very useful, for example to model response styles, see Johnson and Bolt (2010), Wetzel and Carstensen (2017), Plieninger (2016), Henninger and Meiser (2020).

## Mixed Item Formats

Tests often contain a mixture of different item formats. When measuring proficiency, the efficiency of tests can be increased by including binary items, polytomous items, continuous ones as well as count data items. The formats of items in a mixed-format test are often categorized into two classes: multiple choice (MC) and constructed response (CR). As Kim and Lee (2006) noted, typically, MC items are dichotomously scored (DS) and CR items are polytomously scored (PS).

There is a considerable body of methods of scale linking for mixed-format tests. The methods are inspired by the linkage methods for data obtained from two groups of examinees through common items (Kim & Lee, 2006). Common methods are mean/mean, mean/sigma, and Stocking–Lord linkage, see, for example, (Hanson & Béguin, 2002; Ogasawara, 2001; Kim & Hanson, 2002; Kolen & Brennan, 2014).

The thresholds model addresses the problem of different item formats in a quite different way. By construction, it assumes that there is a common latent trait that determines the outcome for all items. The model itself does not distinguish between continuous, polytomous or binary items. The only implicit assumption is that it contains order information.

The difference in item formats is captured in the difficulty functions. They determine which responses can be expected given a fixed ability parameter, and what distributional form the responses have. In the mixed formats case, it is not sensible to assume a common slope in the difficulty functions, instead slopes should vary freely, then item difficulty functions automatically adapt to the item. Resulting item functions can be quite different for, say, a dichotomous item and an item with five categories. The interpretation has to account for the type of item. For the dichotomous item, only the value $\delta (0)$ is relevant, while for a five-categories item the set $\delta (j), j=0,\dots ,4$ determines the response. For continuous functions, the whole difficulty function is interpretable. In contrast to the case of homogeneous item-types, it is less instructive to look at the corresponding item characteristic functions since when considering $P(Y_{pi} > y)$ the value *y* has quite different meaning for different items.

**Fig. 9:**
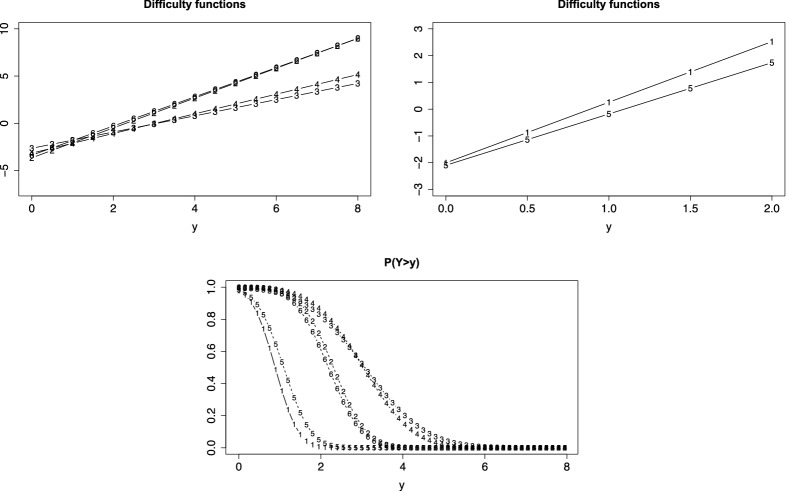
Cognition data with items 1 and 5 as three-categories items. First row: difficulty functions, below: person threshold functions for $\theta =0$

For illustration of mixed item-formats, we consider the cognition data, in which responses range between 1 and 7. We changed the formats of two items, item 1 and item 5, to make them three-categories items by using the thresholds 4 and 6. More precisely, for the items the response is 0 if $$Y_{pi} \le 4$$, 1 if $$ 4 <Y_{pi} \le 6$$, and 2 if $$ Y_{pi} > 6$$. Figure 9 shows the estimated difficulty and PT functions. It is seen that the difficulty functions of the other items (left picture) remain virtually the same as for the original items shown in Fig. 4 (lower right picture). As expected the difficulty functions for items 1 and 5 have changed since now different values of *y* are relevant. Therefore, they are given separately (right picture). Figure 9 also shows the corresponding person thresholds functions. Again the curves for item 1 and item 5 are quite different from the curves in Fig. 4 because for these items the support is different, namely 0,1,2 (corresponding to 0,4,6 on the original *y*-scale), but for the other items the curves are almost the same.

**Fig. 10:**
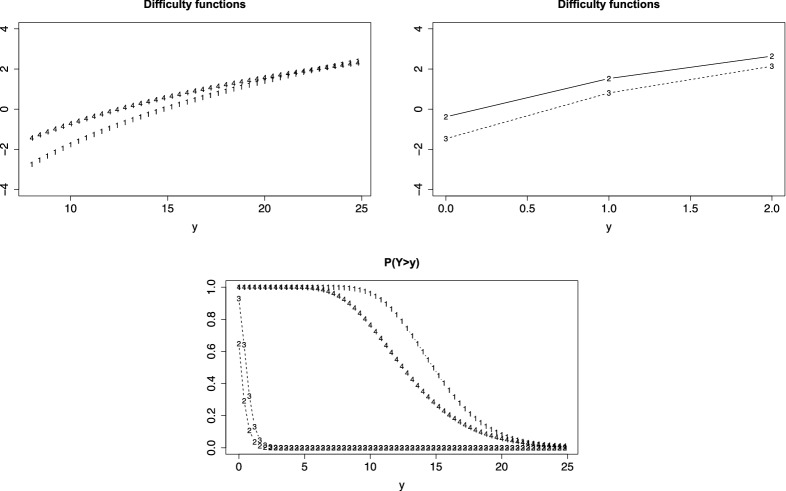
First row: difficulty functions for fluency data with items 2 and 3 changed to three categories items, below: person threshold functions for $\theta =0$

A similar experiment was made for the verbal fluency data which have support $0,1,\dots$. Items 2 and 3 have been changed to three-categories items with support 0,1,2 by using thresholds 9 and 14. Figure 10 shows the difficulty and PT functions for the mixed-formats case. As for the cognition data the PT functions for the unchanged items are very similar to the fits for the original items (see Fig. 7, right picture), but the PT functions for the items 2 and 3 have distinctly changed since the new *y*-values are 0,1,2, which correspond to 0,9,14 on the original response scale.

## More General Models: Flexible Difficulty Functions

The choice of the difficulty function determines the response distribution beyond the choice of the response function. As shown before, it can in particular be used to restrict the support of the response. A fixed choice, for example by using linear difficulty functions, assumes that items differ only by intercepts and slopes (of the difficulty function). A fixed choice has the advantage that each item is determined by just two parameters $\delta _{0i}, \delta _i$, a disadvantage is that the true difficulty function and the distribution of the responses, which depends on the difficulty function, are typically unknown.

A more flexible approach that avoids that one has to choose a specific type of function, and lets the data themselves decide is obtained by letting difficulty functions be determined by basis functions, an approach that has been extensively used in statistics and machine learning (Vidakovic, 1999; Wood, 2006a; b; Ruppert et al. 2009; Wand, 2000). Let us assume that the difficulty functions are given by

3 $$\begin{aligned} \delta _{i}(y) = \sum _{l=0}^M \delta _{il}\Phi _{il}(y), \end{aligned}$$

where $\Phi _{il}(.), l =0,\dots , M$ are chosen basis functions. The simple choice $\Phi _{i0}(y)=1, \Phi _{i1}(y)=y, M=1$ means that the item difficulty functions are linear. Much more flexible models are obtained by alternative functions as radial basis functions or spline functions. A particular attractive choice is B-splines as propagated and motivated extensively by Eilers and Marx (1996, 2021). They are very flexible and can closely approximate a variety of functions. In the literature, they were typically used to approximate functions of observable variables; here they are used to specify the unobservable difficulty functions. If difficulty functions are expanded in basis functions, they have to fulfill that they are non-decreasing, which typically calls for some restrictions. In the case of B-splines, a restriction that ensures that functions are non-decreasing is that $\delta _{i0} \le \dots \le \delta _{iM}$.

If a basis, for example, B-splines have been chosen, there are two basic strategies to select the number of basis functions. One is to choose a relatively small number of basis functions, say 6 or 8, which often is enough to provide the needed flexibility. An alternative strategy is to choose a relatively large number, say 30 to 40 basis functions. Then, the number of coefficients to be estimated increases strongly, and simple log-likelihood fitting is no longer appropriate since it typically yields overfitting. Instead of fitting by maximizing the log-likelihood, one has to use penalization methods, that is, one maximizes a penalized log-likelihood, in which the differences between coefficients of adjacent basis functions are restricted to not vary too strongly, see Eilers and Marx (1996, 2021). Disadvantages are that one has to choose a tuning parameter, for example, by cross-validation, and that one has to deal with a penalized log-likelihood instead of the usual likelihood. Therefore, we use the former strategy in the examples. There is one case where penalization can not be avoided, namely when fitting flexible functions that are supposed to be the same for all items (see Sect. 6). For more general regularization methods that could be adapted to the smoothing of difficulty functions, see also Eilers and Marx (1996), Hastie and Tibshirani 1986) and Bühlmann and Van De Geer (2011).

If difficulty functions have the form (3), the model is parametrized by $\alpha _i,\delta _{i1},\dots ,\delta _{iM}$, $i=1,\dots ,I$, as item parameters, and $\theta _p$, $p=1,\dots ,P$, as person parameters.

Although computation is more demanding, flexible difficulties can be used as a diagnostic tool to investigate if a fixed difficulty function is appropriate. It can also be used to investigate if single items have quite different distributions. One should distinguish between two cases, difficulty function as specified in Eq. (3), which vary freely across items, and a slightly more restrictive approach, which assumes that only the location varies across items. The latter uses the simpler expansion

4 $$\begin{aligned} \delta _{i}(y) = \delta _{0i}+ \sum _{l=0}^M \delta _{l}\Phi _{l}(y). \end{aligned}$$

It assumes that the location $\delta _{0i}$ is item-specific, but the form of the function is the same for all items.

**Fig. 11:**
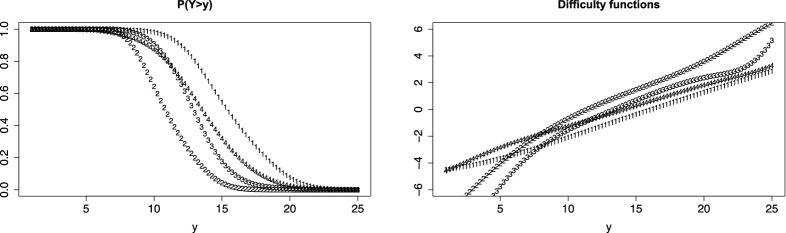
Fitted PT (left) and difficulty (right) functions with B-spline-based difficulty functions for fluency data

Figure 11 shows the PT functions and the fitted difficulty functions for the verbal fluency data if difficulties are not restricted (6 cubic spline functions). It is seen that the obtained PT functions are very similar to the functions obtained for fixed logarithmic difficulty functions (Fig. 7, right picture). The difficulty functions deviate somewhat from logarithmic functions but are not far away in the middle range where observations are located (not shown). Nevertheless, the AIC criterion suggests that the more flexible model should be preferred (4039.59 for the splines fit, 4094.11 for the model with logarithmic difficulty functions). However, the correlation between posterior estimates of person parameters obtained for the splines and the logarithmic model was 0.991, and estimates were very similar. Thus, one might conclude that there is no substantial improvement over the model with logarithmic difficulty functions.

**Fig. 12:**
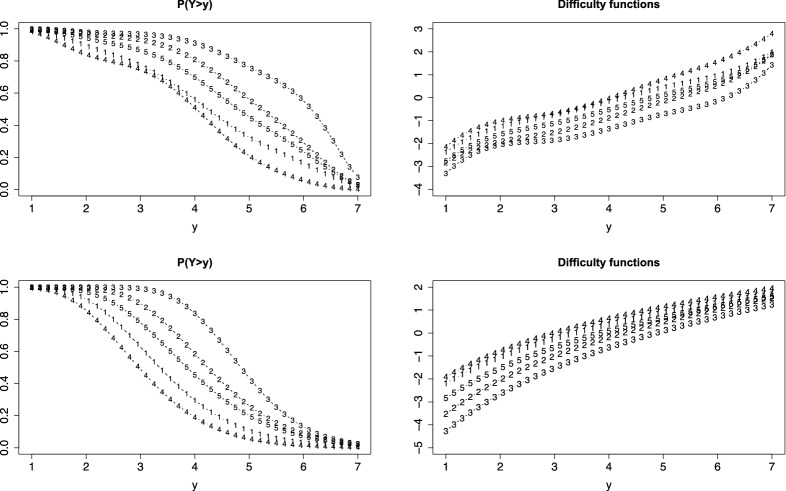
Fitted PT (left) and difficulty (right) functions with B-spline-based difficulty functions for fear data; for comparison second row shows the difficulty functions for logarithmic difficulty functions

Figure 12 shows the PT functions and the fitted difficulty functions for the fear data if B-splines (6 cubic spline functions) generate the difficulty functions, and a discrete distribution is used. For comparison, the second row shows the PT and difficulty functions for logarithmic difficulty functions. Though the order of the items remains the same, the form of the difficulty functions changes if splines are used instead of the logarithmic function. Also the AIC (3484.07) is distinctly smaller than the value obtained for logarithmic difficulty functions (3980.79). The correlation between posterior estimates of person parameters obtained for the splines and the logarithmic model was 0.959, and therefore smaller than for the fluency data.

**Fig. 13:**
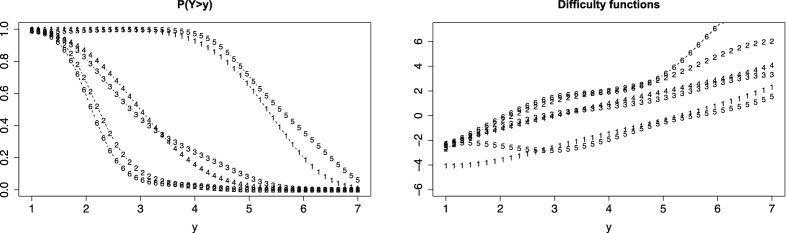
Fitted PT (left) and difficulty (right) functions with B-spline-based difficulty functions for cognition data

Figure 13 shows the PT functions and the fitted difficulty functions for the cognition data if B-splines (6 cubic spline functions) generate the difficulty functions. It is seen that difficulty functions differ from functions obtained for linear functions (see Fig. 4) though the grouping in pairs of items is quite similar. It suggests that the response distributions deviate from the normal distribution, which is implicitly assumed by using linear difficulty functions. AIC for splines was 2803.62, for varying coefficients with fixed difficulty function was 2917.66, the correlation between posterior estimates of person parameters obtained for the splines and the fixed model was 0.956.

## Obtaining Estimates and Inference

In the following, marginal maximum likelihood methods for the estimation of item parameters and posterior estimation of person parameters are considered under the usual assumption of conditional independence of observable variables given the latent variables.

### Marginal Maximum Likelihood Estimation

Let the general thresholds model hold. Then, the distribution function for observation $Y_{pi}$ has the form:

$$\begin{aligned} F_{pi}(y) =P(Y_{pi} \le y) = 1-F( \alpha _i(\theta _p-\delta _{i}(y)) ). \end{aligned}$$

For *continuous* responses, one obtains the density by building derivatives yielding

$$\begin{aligned} f_{pi}(y)=\frac{\partial F_{pi}(y)}{\partial y} = f( \alpha _i(\theta _p-\delta _{i}(y)) ) \alpha _i \delta _{i}'(y), \end{aligned}$$

where *f*(.) is the density corresponding to *F*(.), and $\delta _{i}'(y)= \partial \delta _{i}(y)/\partial y$ is the derivative of the threshold function.

For *discrete* responses $Y_{pi} \in \{0,1,\dots \}$, the probability mass function is obtained by building differences. Then, one has the discrete density function

$$\begin{aligned} f_{pi}(0)&= 1-P(Y_{pi}> 0)=1-F( \alpha _i(\theta _p-\delta _{i}(0)) ),\\ f_{pi}(r)&= P(Y_{pi}> r-1)- P(Y_{pi} > r)\\&=F( \alpha _i(\theta _p-\delta _{i}(r-1)) )-F( \alpha _i(\theta _p-\delta _{i}(r)) ), r=1,2,\dots \end{aligned}$$

where $\sum _r f_{pi}(r)=1$. For simple binary responses, one obtains

$$\begin{aligned} f_{pi}(0)= 1-F( \alpha _i(\theta _p-\delta _{i}(0)) )\quad f_{pi}(1)= F( \alpha _i(\theta _p-\delta _{i}(0)) ), \end{aligned}$$

where $\delta _{0i}=\delta _{i}(0)$ is the familiar difficulty parameter.

If difficulties are expanded in basis functions, they have the form:

$$\begin{aligned} \delta _{i}(y) = \sum _{l=0}^M \delta _{il}\Phi _{il}(y)= {\varvec{\Phi }}_{i}(y)^T\varvec{\delta }_{i}, \end{aligned}$$

where ${\varvec{\Phi }}_{i}(y)^T=(\Phi _{i0}(y),\dots ,\Phi _{iM}(y))$, $\varvec{\delta }_{i}^T=(\delta _{i0},\dots ,\delta _{iM})$. The corresponding derivative is given by

$$\begin{aligned} \delta _{i}'(y) = \sum _{l=0}^M \delta _{il}\Phi _{il}'(y)= {\varvec{\Phi }}_{i}'(y)^T\varvec{\delta }_{i}, \end{aligned}$$

where ${\varvec{\Phi }}_{i}'(y)^T=(\Phi _{i0}'(y),\dots ,\Phi _{iM}'(y))$ is the vector of derivatives of basis functions.

Let now observations be given by $y_{pi}, i=1,\dots ,I, p=1,\dots ,P$. The estimation method that is used is marginal likelihood by assuming that person parameters are normally distributed, $\theta _p\sim N({0}, \sigma _{\theta }^2)$. Maximization of the marginal log-likelihood can be obtained by integration techniques. We use numerical integration by Gauss–Hermite integration methods. Early versions for univariate random effects date back to Hinde (1982) and Anderson and Aitkin (1985).

Let $\varvec{\delta }_{i}$ denote the vector of all parameters linked to the difficulty function of item *i*. For fixed difficulty functions, it has length two, for expansions in basis functions it is, more generally, $M+1$. The vector $\varvec{\alpha }^T=(\alpha _1,\dots ,\alpha _I)$ collects the item slopes. With $\varvec{\delta }^T=(\varvec{\delta }_{1}^T,\dots ,\varvec{\delta }_{I}^T,\varvec{\alpha }^T,\sigma _{\theta })$ denoting the set of all item parameters and $f_{0,\sigma _{\theta }}(.)$ denoting the density of the normal distribution $N({0}, \sigma _{\theta }^2)$, the marginal likelihood has the form:

$$\begin{aligned} L(\varvec{\delta })=\prod _{p=1}^P \int \prod _{i=1}^I f_{pi}(y_{pi}) f_{0,\sigma _{\theta }}(\theta _p) \mathrm{d}\theta _p, \end{aligned}$$

yielding the log-likelihood

$$\begin{aligned} l(\varvec{\delta })= \log (L(\varvec{\delta })) = \sum _{p=1}^P \log \left( \int \prod _{i=1}^I f_{pi}(y_{pi}) f_{0,\sigma _{\theta }}(\theta _p) \mathrm{d}\theta _p\right) . \end{aligned}$$

The score function $s(\varvec{\delta }) = \partial l/ \partial \varvec{\delta }$, which takes on the value of 0 for maximum likelihood estimates, has components

$$\begin{aligned} \frac{\partial l}{ \partial \delta _{ij}}&= \sum _{p=1}^P \int \frac{ \partial f_{pi}(y_{pi})}{ \partial \delta _{ij}} \prod _{l \ne i}f_{pi}(y_{pl})f_{0,\sigma _{\theta }}(\theta _p) \mathrm{d}\theta _p / c_p,\\ \frac{\partial l}{ \partial \alpha _{i}}&= \sum _{p=1}^P \int \frac{ \partial f_{pi}(y_{pi})}{ \partial \alpha _{i}} \prod _{l \ne i}f_{pi}(y_{pl})f_{0,\sigma _{\theta }}(\theta _p) \mathrm{d}\theta _p / c_p,\\ \frac{\partial l}{ \partial \sigma _{\theta }}&= \sum _{p=1}^P \int \prod _{i=1}^I f_{pi}(y_{pi}) \frac{\partial f_{0,\sigma _{\theta }}(\theta _p)}{\partial \sigma _{\theta }} \mathrm{d}\theta _p/ c_p, \end{aligned}$$

where $c_p = \int \prod _{i=1}^I f_{pi}(y_{pi}) f_{0,\sigma _{\theta }}(\theta _p) \mathrm{d}\theta _p$. The derivation uses that the order of integration and differentiation can be interchanged, which holds if densities are continuous and continuously differentiable, in particular it holds if *F*(.) is the normal distribution function.

The form of the derivatives depends on the distribution of the responses. For continuous responses, one obtains

$$\begin{aligned} \frac{ \partial f_{pi}(y_{pi})}{ \partial \delta _{ij}}&= \alpha _{i}\Phi _{ij}'(y_{pi})f(\alpha _{i}(\theta _p-\delta _{i}(y_{pi})))-\alpha _{i}^2 f'(\alpha _{i}(\theta _p-\delta _{i}(y_{pi})))\Phi _{ij}(y_{pi}){\varvec{\Phi }}_{i}'(y_{pi})^T\varvec{\delta }_{i},\\ \frac{ \partial f_{pi}(y_{pi})}{ \partial \alpha _{i}}&= {\varvec{\Phi }}_{i}'(y_{pi})^T\varvec{\delta }_{i}\{f(\alpha _{i}(\theta _p-\delta _{i}(y_{pi}))+ \alpha _{i}(\theta _p-{\varvec{\Phi }}_{i}(y_{pi})^T\varvec{\delta }_{i})f'(\alpha _{i}(\theta _p-\delta _{i}(y_{pi})))\}, \end{aligned}$$

with $f'(.)$ denoting the derivative of *f*(.). For discrete responses, one has

$$\begin{aligned} \frac{ \partial f_{pi}(y_{pi})}{ \partial \delta _{ij}}&= -\alpha _{i} f(\alpha _{i}(\theta _p-\delta _{i}(y_{pi}-1)))\Phi _{ij}(y_{pi}-1)+ \alpha _{i} f(\alpha _{i}(\theta _p-\delta _{i}(y_{pi})))\Phi _{ij}(y_{pi}),\\ \frac{ \partial f_{pi}(y_{pi})}{ \partial \alpha _{i}}&= (\theta _p-\delta _{i}(y_{pi}-1)) f(\alpha _{i}(\theta _p-\delta _{i}(y_{pi}-1)))- (\theta _p-\delta _{i}(y_{pi})) f(\alpha _{i}(\theta _p-\delta _{i}(y_{pi}))), \end{aligned}$$

where $\Phi _{ij}(-1)$ is defined by $\Phi _{ij}(-1)=0$ and $\delta _{i}(-1)= -\infty$.

For simple difficulty functions, the score functions simplify accordingly. For example, when the difficulty functions are linear, one has ${\varvec{\Phi }}_{i}(y)^T=(1,y)$, and ${\varvec{\Phi }}_{i}'(y)^T=(0,1)$. An approximation of the covariance of the estimate, ${\mathrm{cov}}(\hat{\varvec{\delta }})$, is obtained by the observed information $-\partial ^2 l/ \partial \varvec{\delta }\partial \varvec{\delta }^T$.

Some caution is needed when fitting the model (4) with a common difficulty function expanded in B-splines. Since B-splines sum up to 1 at any given value, the parameters in model (4) are not identified. This can be fixed by choosing a fixed value for one of the parameters $\delta _{0i}$, for example, $\delta _{01}=0$. One can also use the general form (3) and use a tailored penalty. Instead of maximizing the log-likelihood, one maximizes the penalized log-likelihood $l(\{\delta _i\})= l(\{\delta _i\}) - P_{\lambda }(\{\delta _i\})$ with penalty term

$$\begin{aligned} P_{\lambda }(\{\delta _i\}) = \lambda \sum _{i=2}^I \sum _{l=2}^M [(\delta _{il}-\delta _{i,l-1})- (\delta _{i-1,l}-\delta _{i-1,l-1})]^2. \end{aligned}$$

The choice of $\lambda$ determines the estimates. For $\lambda =0$, one maximizes the usual log-likelihood. For $\lambda \longrightarrow \infty$, the differences of adjacent parameters become the same for all items, that is, $\delta _{il}-\delta _{i,l-1}=\delta _{i-1,l}-\delta _{i-1,l-1}$, otherwise the penalty term would also go to infinity. Then, the levels of the functions can differ but not the form of the function. Therefore, using a large value of $\lambda$ automatically yields shifted difficulty functions.

For the computation of estimates, we used Gauss–Hermite integration, which works rather well since all integrals are unidimensional. The written R program uses the computation of derivatives in combination with the R function *optim*.

### Illustrative Simulation

**Fig. 14:**
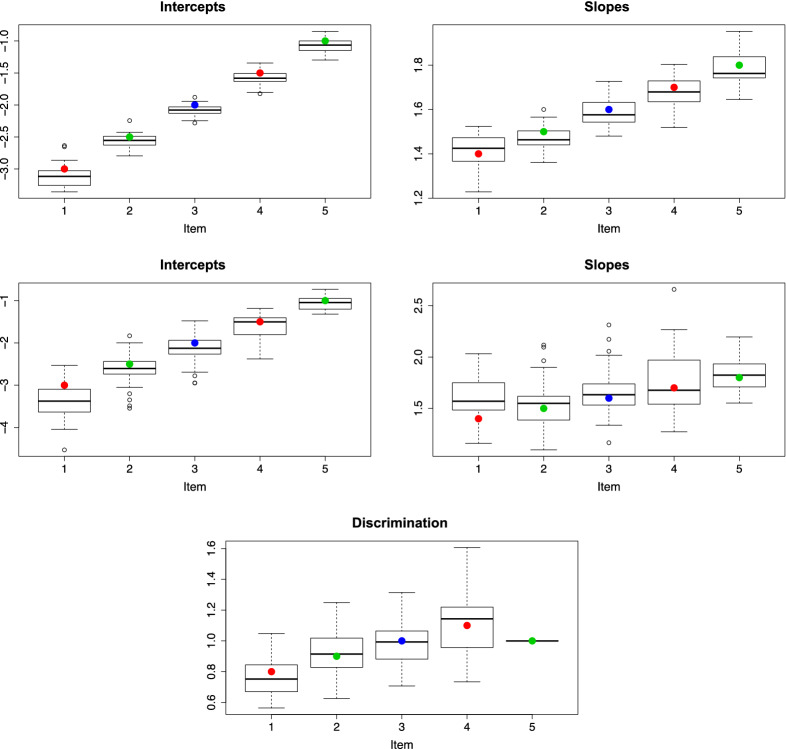
Estimates for simulated count data; first row: fixed discrimination parameters ($\alpha _i=1$); second and third row: varying discrimination parameters, which are also estimated

For illustration, we show the results of a small simulation study. Figure 14 shows the estimates for count data with varying slopes for $I=5$, $P=200$ and $\sigma _{\theta }=1$. The dots show the true values of the parameters. The first row shows estimates if there is no variation in discrimination parameters, $\alpha _i=1$, which is also assumed when estimating parameters. The second and third row shows estimates for varying discrimination parameters. It is seen that the parameters are estimated rather well if discrimination parameters are fixed. If they are varying, estimation becomes less accurate but is able to separate discrimination parameters from intercepts and slopes. There is no variation in the last discrimination parameter since it was fixed ($\alpha _5=1$).

### Estimating Person Parameters

If estimates of item parameter are found, posterior mode or mean estimation yields estimates of person parameters. For given item responses $\varvec{y}^T=(y_1,\dots ,y_I)$, the posterior is given by

$$\begin{aligned} f(\theta |\varvec{y}, \varvec{\delta }) = \frac{\prod _{i=1}^I f(\alpha _{i}(\theta _p-\delta _{i}(y_i)))\alpha _{i}\delta _{i}'(y_i) f_{0,\sigma _{\theta }}(\theta _p)}{\int \prod _{i=1}^I f(\alpha _{i}(\theta _p-\delta _{i}(y_i)))\alpha _{i}\delta _{i}'(y_i) f_{0,\sigma _{\theta }}(\theta _p) \mathrm{d}\theta _p}. \end{aligned}$$

Replacing the parameter $\varvec{\delta }$ by its estimate $\hat{\varvec{\delta }}$ allows to compute the mode of the posterior $\hat{\theta }_m$ or the posterior mean

$$\begin{aligned} \hat{\theta }_{m}={\text {E}}(\theta |\varvec{y}, \varvec{\delta }) = \int \theta f(\theta |\varvec{y}, \varvec{\delta }) \mathrm{d}\theta . \end{aligned}$$

**Fig. 15:**
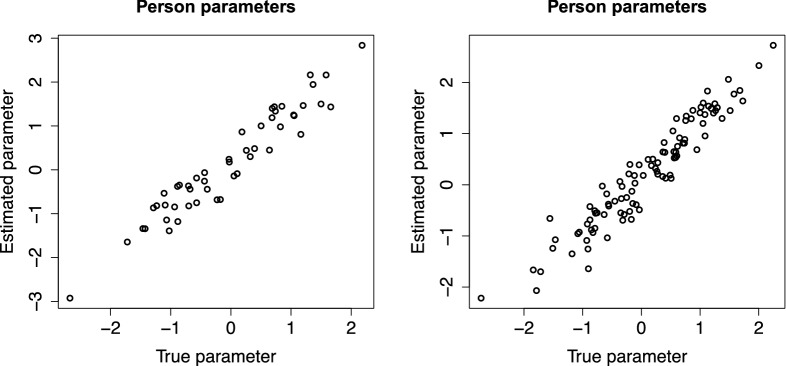
True person parameters plotted against fitted values for simulation data for P=50 (left) and P=100 (right)

Figure 15 illustrates that posterior estimates are close to true values. Data were generated for 10 items with linear difficulty functions. The intercepts were chosen as $\delta _{i0}= -2.25 +(i-1)0.5$, $i=1,\dots ,10$, yielding $-2.25, -1.75, \dots , 2.25$. The slopes of the first four items were $\delta _{i}=1$, for the next four items $\delta _{i}=2$, and for the remaining two items $\delta _{i}=3$. For $P= 50$ and $P=100$ with $\theta _p$ drawn randomly from *N*(0, 1), one obtains the plots of true person parameters against estimated parameters shown in Fig. 15.

## Concluding Remarks

The comprehensive class of thresholds models has been introduced and illustrated in examples. Also basic properties of the model class have been shown. Future research might be devoted to extensions of the model class and further investigations of its properties. As already mentioned, it is straightforward to include explanatory variables by using the additive term $\theta _p +\varvec{x}_p^T{\varvec{\beta }}- \delta _{i}(y)$ instead of the simple term $\theta _p -\delta _{i}(y)$, where $\varvec{x}_p$ is a person-specific explanatory variable and ${\varvec{\beta }}$ the corresponding weight. The latter can also be item-specific. The incorporation of explanatory variables can be useful to investigate sources of heterogeneity in a response scale, and has been propagated, for example, by Jeon and De Boeck (2016). Also the extension to multidimensional models is a possible topic of further research. Various general methods of model checking for categorical responses have been proposed (Swaminathan et al., 2006; Maydeu-Olivares, 2013; Haberman et al., 2013). They can also be applied to thresholds models, which, in the case of categorical responses are specific graded response models. Similar approaches might be developed for continuous responses and count data taking the specific distributions into account. It might also be useful to exploit that all thresholds models become simply structured, familiar binary models if responses are dichotomized. Then, model checking for binary models can be used, but one has to find ways how to combine the results obtained for the dichotomizations.

We restricted consideration to symmetric response functions *F*(.). The use of a normal or a logistic response function yields very similar results, although the scaling is different. However, the use of non-symmetric distributions as, for example, the extreme value distribution might make a difference. In principle also discrete response functions could be used, the extreme case being a zero-one function as in the Guttman model; however, they include jumps that might be less realistic when assuming a continuous latent trait.

Software for the computation of marginal maximum likelihood estimates will be made available on Github.

### Supplementary Information

Below is the link to the electronic supplementary material.

Supplementary file 1 (pdf 0 KB)

## Appendix

### Proposition 8.1

If the item difficulty function in the thresholds model with continuous distribution function *F*(.) and corresponding density $f(y) = \partial F(y)/\partial y$ is linear, $\delta _{i}(y)= \delta _{0i}+ \delta _i y$, $\delta _i \ge 0$ one obtains for the expectation and the variance

5 $$\begin{aligned}&\mu _{pi}={\text {E}}(Y_{pi}) = (\theta _p - \delta _{0i} - E_F/\alpha _i)/\delta _i, \end{aligned}$$

6 $$\begin{aligned}&\sigma _{pi}^2={\text {var}}(Y_{pi}) = var_F /(\alpha _i\delta _i)^2, \end{aligned}$$

where $E_F=\int y f(y)\mathrm{d}y$ is the expectation corresponding to distribution function *F*(.), and $var_F =\sigma _F^2=\int (y-E_F)^2f(y)d y$ is the variance linked to *F*(.)

If, in addition, *F*(.) is symmetric the distribution function of $Y_{pi}$ is a shifted and scaled version of *F*(.) and the density is given by $\sigma _F f(\sigma _F(y-\mu _{pi})/\sigma _{pi})/\sigma _{pi}$, which simplifies to $f((y-\mu _{pi})/\sigma _{pi})/\sigma _{pi}$ if $\sigma _F=1$.

### Proof

For linear item function, the thresholds model has the form $P(Y_{pi} > y|\theta _p,\alpha _i,\delta _{i}(.))=F( \alpha _i(\theta _p-\delta _{0i}- \delta _i y) )$. The corresponding distribution function is

$$\begin{aligned} F_{Y_{pi}}(y) = P(Y_{pi} \le y) = 1 - F(\alpha _i(\theta _p-\delta _{0i}- \delta _i y)). \end{aligned}$$

The density is given by

$$\begin{aligned} f_{Y_{pi}}(y)=\frac{\partial F_{Y_{pi}}(y)}{\partial y} = f(\alpha _i(\theta _p-\delta _{0i}- \delta _i y))\alpha _i\delta _i, \end{aligned}$$

yielding the expectation

$$\begin{aligned} {\text {E}}(Y_{pi})= \alpha _i\delta _i \int y f(\alpha _i(\theta _p-\delta _{0i}- \delta _i y)) \mathrm{d}y. \end{aligned}$$

With $\eta = \alpha _i(\theta _p-\delta _{0i}- \delta _i y)$ and $\mathrm{d}\eta /\mathrm{d}y=- \alpha _i\delta _i$ one obtains

$$\begin{aligned} {\text {E}}(Y_{pi})= -\int _{\infty }^{-\infty } \frac{\theta _p-\delta _{0i}-\eta /\alpha _i}{\delta _i} f(\eta ) \mathrm{d}\eta = \frac{\theta _p-\delta _{0i}-E_F/\alpha _i}{\delta _i} \end{aligned}$$

where $E_F=\int y f(y)\mathrm{d}y$ is a parameter that depends on *F* but not on *i*.

The variance is given by

$$\begin{aligned} {\text {var}}(Y_{pi})&= \int \left( y-\frac{\theta _p-\delta _{0i}-E_F/\alpha _i}{\delta _i}\right) ^2 f(\alpha _i(\theta _p-\delta _{0i}- \delta _i y))\alpha _i\delta _i \mathrm{d}y = \int (\frac{\eta -E_F}{\alpha _i\delta _i})^2 f(\eta ) \mathrm{d}\eta \\&= {\text {var}}_F /(\alpha _i^2{\delta _i}^2), \end{aligned}$$

where $${\text {var}}_F=\int ({\eta -E_F})^2 f(\eta ) \mathrm{d}\eta $$.

If *F*(.) is symmetric, $E_F=0$ and therefore $\mu _{pi}=(\theta _p-\delta _{0i})/\delta _i$. With $\sigma _F=\sqrt{{\text {var}}_F}$, one obtains

$$\begin{aligned} f_{Y_{pi}}(y)= f(\alpha _i(\theta _p-\delta _{0i}- \delta _i y))\alpha _i\delta _i= f(\alpha _i\delta _i(\mu _{pi}-y))\alpha _i\delta _i=\sigma _F f(y-\mu _{pi})/\sigma _{pi})/\sigma _{pi}. \end{aligned}$$

For standardized response function ($\sigma _F=1$), one obtains the simple form $f_{Y_{pi}}(y)= f((y-\mu _{pi})/\sigma _{pi})/\sigma _{pi}$. $\square$

### Proposition 8.2

For the thresholds model with continuous response function *F*(.), let the difficulty functions be defined on the support *S* taking values from $\mathbb {R} \cup \{\infty \}$.

1. Person parameters and item functions are identifiable if one discrimination parameter and one person parameter are fixed, for example, $\alpha _1=1,\theta _1=0$.
2. Person parameters and item functions are identifiable if one discrimination parameter is fixed, for example $\alpha _1=1$, and $\delta _i (y_0)$ is fixed for one value $y_0 \in \tilde{S}$, where $\tilde{S}=S$ if *S* is infinite and $\tilde{S}=\{m_1,\dots , m_{k-1}\}$ for finite support $S=\{m_1,\dots , m_k\}$. In the latter case, $\delta _i (m_k)=\infty$ holds for all items.

### Proof

Since the response function is strictly increasing, one has

7 $$\begin{aligned} \alpha _i(\theta _p- \delta _i(y)) = \tilde{\alpha }_i(\tilde{\theta }_p- \tilde{\delta }_i(y)) \end{aligned}$$

for all items and values $y \in S$.

Let now for two parameterizations $\theta _p,\delta _i (.)$ and $\tilde{\theta }_p,\tilde{\delta }_i (.)$ one discrimination parameter and one person parameter be fixed by $\alpha _1=\tilde{\alpha }_1=1,\theta _1=\tilde{\theta }_1=0$. If one chooses $\theta _1=0$, and accordingly $\tilde{\theta }_1=0$ one obtains $\alpha _i\delta _i(y)=\tilde{\alpha }_i\tilde{\delta }_i(y)$ for all items and values $y \in S$. For item 1 one obtains $\delta _1(y)=\tilde{\delta }_1(y)$, and therefore $\theta _p=\tilde{\theta }_p$. Using $\tilde{\delta }_i(y)=(\alpha _i/\tilde{\alpha }_i)\delta _i(y)$ in (7) yields $\alpha _i\theta _p=\tilde{\alpha }_i\tilde{\theta }_p$ and therefore $\alpha _i =\tilde{\alpha }_i$, from which $\delta _i(y)=\tilde{\delta }_i(y)$ follows.

Let now $\alpha _1=\tilde{\alpha }_1=1$ and $\delta _i (y_0)=\tilde{\delta }_i (y_0)=0$ for one value $y_0 \in S$ for infinite support *S*. Then, one obtains from (7) for item 1 and $y=y_0$ that $\theta _p = \tilde{\theta }_p$ holds for all persons. Equation (7) yields $(\alpha _i-\tilde{\alpha }_i)\theta _p=\alpha _i\delta _i(y)-\tilde{\alpha }_i\tilde{\delta }_i(y)$, which for $y=y_0$ yields $(\alpha _i-\tilde{\alpha }_i)\theta _p=0$ and therefore $\alpha _i=\tilde{\alpha }_i$. Then, one also has $\delta _i(y)=\tilde{\delta }_i(y)$ .

If the support is finite, one has to choose $\delta _i (y_0)=0$ for $y_0 \in \{m_1,\dots , m_{k-1}\}$ since for $\delta _i (m_k)$ one always has $\delta _i (m_k)=\infty$ because $0=P(Y_{pi}>m_k)=F(\theta _p- \delta _i(m_k))$ has to hold for any $\theta _p$. If $\delta _i (y_0)=0$ is chosen accordingly (and $\alpha _1=\tilde{\alpha }_1=1$), the derivation is the same as in the case of infinite support. $\square$

### Proposition 8.3

The thresholds model $P(Y_{pi} > y)=F( \alpha _i(\theta _p-\delta _{i}(y)) )$ holds for continuous response $Y_{pi}$ iff the graded response model holds for all categorizations

$$\begin{aligned} Y_{pi}^{(c)} =r \quad \Longleftrightarrow \quad Y_{pi} \in (\tau _{r}, \tau _{r+1}], \end{aligned}$$

where $\tau _{1}< \dots < \tau _{k}$ are any ordered thresholds.

### Proof

Let the thresholds model $P(Y_{pi} > y|\theta _p,\alpha _i,\delta _{i}(.))=F( \alpha _i(\theta _p-\delta _{i}(y)) )$ hold for continuous response $Y_{pi}$ for some increasing function $\delta _{i}(y)$. Let a categorized version be defined by

$$\begin{aligned} Y_{pi}^{(c)} =r \text { if } Y_{pi} \in (\tau _{r}, \tau _{r+1}]. \end{aligned}$$

for any partition $\tau _{0}= -\infty< \tau _{1}< \dots < \tau _{k}$.

One obtains $P(Y_{pi} > \tau _{r}|\theta _p,\alpha _i,\delta _{i}(.))=F( \alpha _i(\theta _p-\delta _{i}(\tau _{r})) )$ and therefore with $\delta _{ir}=\delta _{i}(\tau _{r})$

8 $$\begin{aligned} P(Y_{pi}^{(c)} \ge r) =F( \alpha _i(\theta _p-\delta _{ir})) , \end{aligned}$$

which is the graded response model for discrete response $Y_{pi}^{(c)}$.

Let now the discretized version (8) hold for all discretizations. Let us consider the discretization $\tau _{1}< \dots < \tau _{k}$ with response $Y_{pi}^{(c)}$ and parameters $\delta _{ir}$, and the discretization $\tau _{1}+\Delta< \dots < \tau _{k}$ with response $Y_{pi}^{(c_{\Delta })}$ and parameters $\delta _{ir}^{(c_{\Delta })}$, where $\Delta < \tau _{2}-\tau _{1}$. Let the difficulty function be defined by $\delta _i(\tau _{1}) = \delta _{i1}$, $\delta _i(\tau _{1}+\Delta ) = \delta _{i1}^{(c_{\Delta })}$ to obtain

$$\begin{aligned} P(Y_{pi}> \tau _{1})= F( \alpha _i(\theta _p-\delta _i(\tau _{1})) ), \quad P(Y_{pi} > \tau _{1}+\Delta )= F( \alpha _i(\theta _p-\delta _i(\tau _{1}+\Delta )) ), \end{aligned}$$

Since this holds for any values $\tau _{1}, \Delta$ one obtains the thresholds model for continuous response $Y_{pi}$. $\square$
