# Supplementary material for: Item Response Thresholds Models: A General Class of Models for Varying Types of Items
Source: Psychometrika. 2022 Apr 27;87(4):1238–69. doi: 10.1007/s11336-022-09865-7 (PMC9636304; doi:10.1007/s11336-022-09865-7)
Supplement: Supplementary file 1 — (pdf 0 KB) [file 11336_2022_9865_MOESM1_ESM.pdf]

Online Appendix

Figure 16 shows the estimates for count data with constant slopes and varying discrimination parameters for $I = 5$, $P = 200$ and $\sigma_\theta = 1$. The dots show the true values of the parameters. Note that there is no variation in the last discrimination parameter since it is fixed ($\alpha_5 = 1$). Figure 17 shows the estimates for data with varying slopes and fixed discrimination parameters ($I = 5$, $P = 200$, $\sigma_\theta = 1$). For illustration Figure 18 shows the estimates for the latter scenario if the number of persons is increased to $P = 400$, which means estimates are closer to the true values.

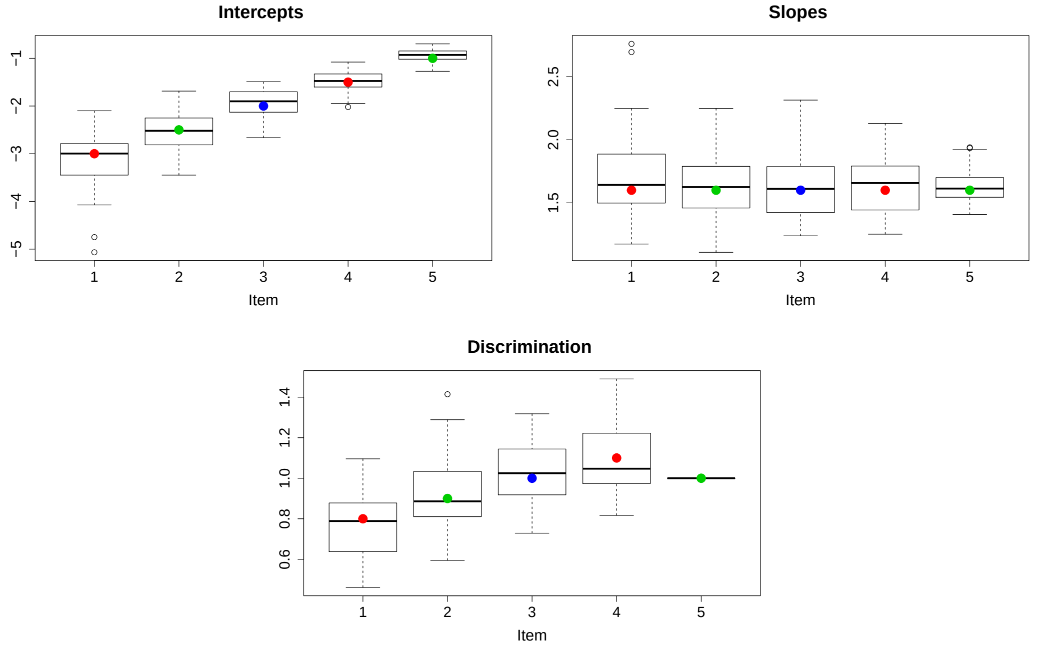

FIGURE 16: *Estimates for simulated count data with $I = 5$, $P = 200$ and $\sigma_\theta = 1$. The dots show the true values of the parameters.*

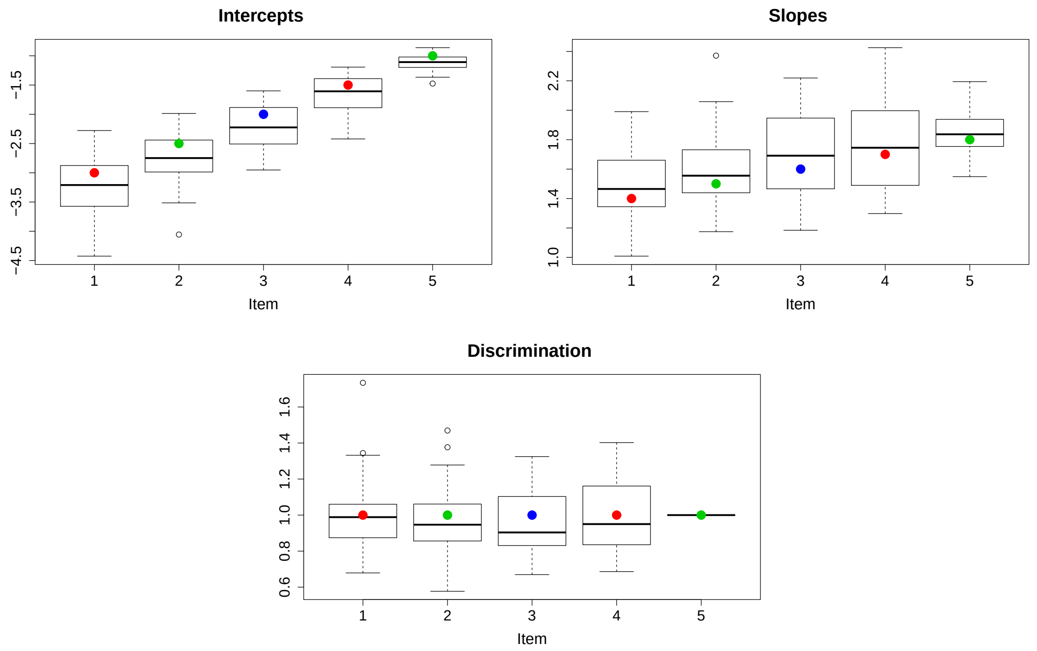

FIGURE 17: *Estimates for simulated count data with* $I = 5$, $P = 200$ *and* $\sigma_\theta = 1$. *The dots show the true values of the parameters.*

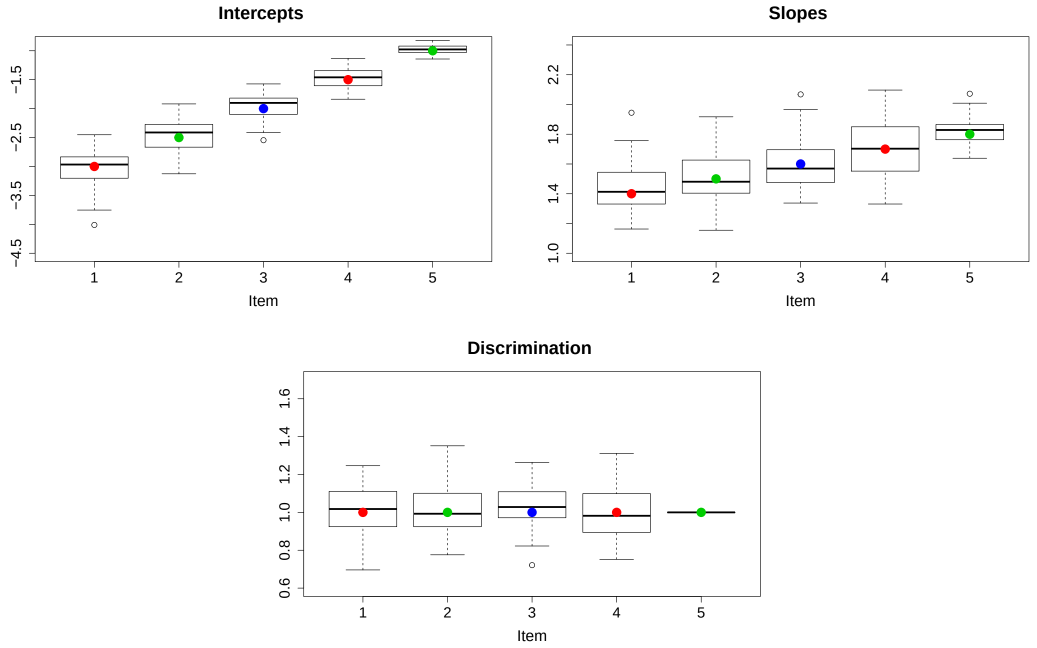

FIGURE 18: *Estimates for simulated count data with* $I = 5$, $P = 400$ *and* $\sigma_\theta = 1$. *The dots show the true values of the parameters.*
